# Understanding the Chemical Mechanism behind Photoinduced Enhanced Raman Spectroscopy

**DOI:** 10.1021/acs.jpclett.3c00478

**Published:** 2023-05-11

**Authors:** Junzhi Ye, Rakesh Arul, Michel K. Nieuwoudt, Junzhe Dong, Ting Zhang, Linjie Dai, Neil C. Greenham, Akshay Rao, Robert L. Z. Hoye, Wei Gao, M. Cather Simpson

**Affiliations:** † The Photon Factory, The University of Auckland, Auckland 1010, New Zealand; ‡ Department of Chemical and Materials Engineering, The University of Auckland, Auckland 1010, New Zealand; § Cavendish Laboratory, University of Cambridge, JJ Thomson Avenue, Cambridge CB3 0HE, United Kingdom; ∥ The MacDiarmid Institute for Advanced Materials and Nanotechnology, Wellington 6012, New Zealand; ⊥ The Dodd Walls Centre for Quantum and Photonic Technologies, Dunedin 9054, New Zealand; # School of Chemical Sciences, The University of Auckland, Auckland 1010, New Zealand; ¶ Department of Physics, The University of Auckland, Auckland 1010, New Zealand; ∇ Inorganic Chemistry Laboratory, University of Oxford, South Parks Road, Oxford OX1 3QR, United Kingdom

## Abstract

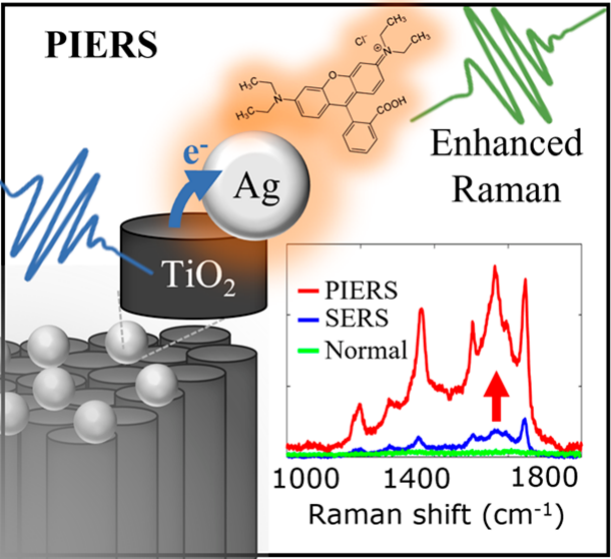

Photoinduced enhanced Raman spectroscopy (PIERS) is a new surface enhanced Raman spectroscopy (SERS) modality with a 680% Raman signal enhancement of adsorbed analytes over that of SERS. Despite the explosion in recent demonstrations, the PIERS mechanism remains undetermined. Using X-ray and time-resolved optical spectroscopies, electron microscopy, cyclic voltammetry, and density functional theory simulations, we elucidate the atomic-scale mechanism behind PIERS. Stable PIERS substrates were fabricated using self-organized arrays of TiO_2_ nanotubes with controlled oxygen vacancy doping and size-controlled silver nanoparticles. The key source of PIERS vs SERS enhancement is an increase in the Raman polarizability of the adsorbed analyte upon photoinduced charge transfer. A balance between improved crystallinity, which enhances charge transfer due to higher electron mobility but decreases light absorption, and increased oxygen vacancy defect concentration, which increases light absorption, is critical. This work enables the rational design of PIERS substrates for sensing.

Raman spectroscopy is a widely used tool in the analytical sciences, enabling highly sensitive fingerprinting of molecules for healthcare, security, and environmental applications. However, Raman scattering is inefficient and only accounts for one in ten million scattered photons.^1^ Surface enhanced Raman spectroscopy (SERS)^2^ uses nanostructured plasmonic metals (e.g., Au, Ag, Al) supporting a localized surface plasmon resonance that enhances the near-field intensity of incident laser light. The enhancement of Raman scattering can be spectacular, with state-of-the-art techniques reaching enhancement factors (EF) as large as 10^9^,^3^ with typical values between 10^4^ and 10^8^.^4^ Recently, the use of dielectrics as support or active materials in SERS has grown. Dielectrics can act as passive elements by supporting plasmonic nanostructures to localize optical fields via a microlensing effect^5^ and provide an inert shell that enables in situ SERS measurements of chemical reactions (e.g., SHINERS^6^ or SPARKs^7^). Pure dielectric particles may also concentrate optical fields but have not achieved single-molecule SERS (typical dielectric SERS EF = 10^2^–10^6^ ^8^) and are not as reproducible as plasmonic SERS.^9^ However, dielectric SERS^10^ remains useful due to its abundance, lower cost, and its ability to interrogate chemical reactions,^11^ complementing emerging plasmonic metals such as Mg^12^ and Al.^13^ Semiconductors may also act as active SERS substrates via charge transfer to an adsorbed molecule^10,14^ and form reusable SERS substrates through photocatalytic degradation of adsorbed molecules^15^ upon exposure to ultraviolet light.

A new development that aims to push the enhancement factor of Raman beyond that of SERS is photoinduced enhanced Raman spectroscopy (PIERS).^17,18^ In PIERS, a defect engineered semiconductor (e.g., TiO_2_) acts in concert with plasmonic nanoparticles to enhance Raman signals by an order-of-magnitude relative to SERS. The commonly proposed enhancement mechanism^17^ is as follows: TiO_2_ containing oxygen vacancies can absorb visible light due to sub-bandgap defects. Once in the conduction band, mobile electrons migrate to the plasmonic nanoparticle, and charge transfer occurs from the metal to the adsorbed molecules. Prior works have proposed a charge-transfer based enhancement for many dye molecules in pure/defect engineered semiconductors,^19−26^ semiconductor heterostructures,^27,28^ or semiconductor–metal heterostructures.^29^ However, such works have used conditions in which the dye molecule (such as Rhodamine-6-G) is on- or near-resonance with the excitation laser,^19,21,24,28,30−34^ thus having resonance Raman enhancements in addition to substrate-induced enhancements. Other works claim charge transfer without providing evidence for the exact mechanism.^22,23,35^ Indeed only a few studies^19,25,29,32,36,37^ show evidence that bona fide charge transfer resonances between localized molecular states to semiconductor bands are boosting the observed Raman signals, while others^38,39^ do not provide any direct evidence of energetically favorable band alignments. No effort has been made yet to map out the molecular and semiconductor energy levels involved to establish the PIERS enhancement mechanism.

Here, we determine the mechanism of PIERS and find that instead of the commonly accepted charge transfer resonance Raman enhancement model proposed previously,^17,26,40^ the most likely enhancement mechanism involves an increase in the intrinsic polarizability of the adsorbed molecule upon charge transfer. We also demonstrate a new method for making stable, reproducible PIERS substrates that utilizes simple annealing, deposition, and self-organization techniques.

Our PIERS substrates are composed of crystalline anodized TiO_2_ nanotube arrays with a surface layer of thermally dewetted plasmonic Ag nanoparticles (AgNPs) and a controlled amount of oxygen vacancy disorder (Figure 1b, fabrication details in methods section and Supporting Information Section I). The equivalent SERS substrate is identical other than using amorphous instead of crystalline TiO_2_ (Figure 1a). The PIERS substrate’s surface atomic structure is a mixture of crystalline anatase and rutile TiO_2_, while the SERS and as-anodized TiO_2_ substrates are amorphous (Figure 1c). The characterization of the PIERS substrate’s structure is supported by high resolution TEM images (Figure 1d) that show plasmonic AgNPs in close contact with the anatase and rutile phases (Figure S7). X-ray diffraction measurements (Figure S8) show a 35% rutile to 65% anatase phase fraction for the PIERS substrate. AgNPs are uniformly distributed on the surface of the TiO_2_ nanotube array and have a bimodal size distribution clustering around 5–15 nm and 65–75 nm (Supporting Information Figure S4). However, not all AgNPs contribute to the SERS signal due to the size-dependence of the plasmonic resonance. The particle density of AgNPs resonant with the 488 nm Raman excitation laser is consistent between the SERS and PIERS substrates, and many plasmonic hotspots are excited by the several μm diameter Raman laser probe. By varying the TiO_2_ annealing temperature, annealing environment, dewetting temperature, and the thickness of the magnetron-sputtered Ag layer, we engineer a large Raman signal enhancement, suitable plasmon resonance, and high degree of uniformity over the surface (Table S2, Supporting Information). The advantage of our fabrication approach over the original realization of PIERS^17,26^ is the presence of air-stable oxygen vacancy defects^41^ within TiO_2_ created through thermal methods rather than UV irradiation which creates short-lived defects,^26,42^ and a simple self-assembly strategy to create AgNPs via the templating effect of the well-ordered TiO2 nanotube array beneath.

**Figure 1:**
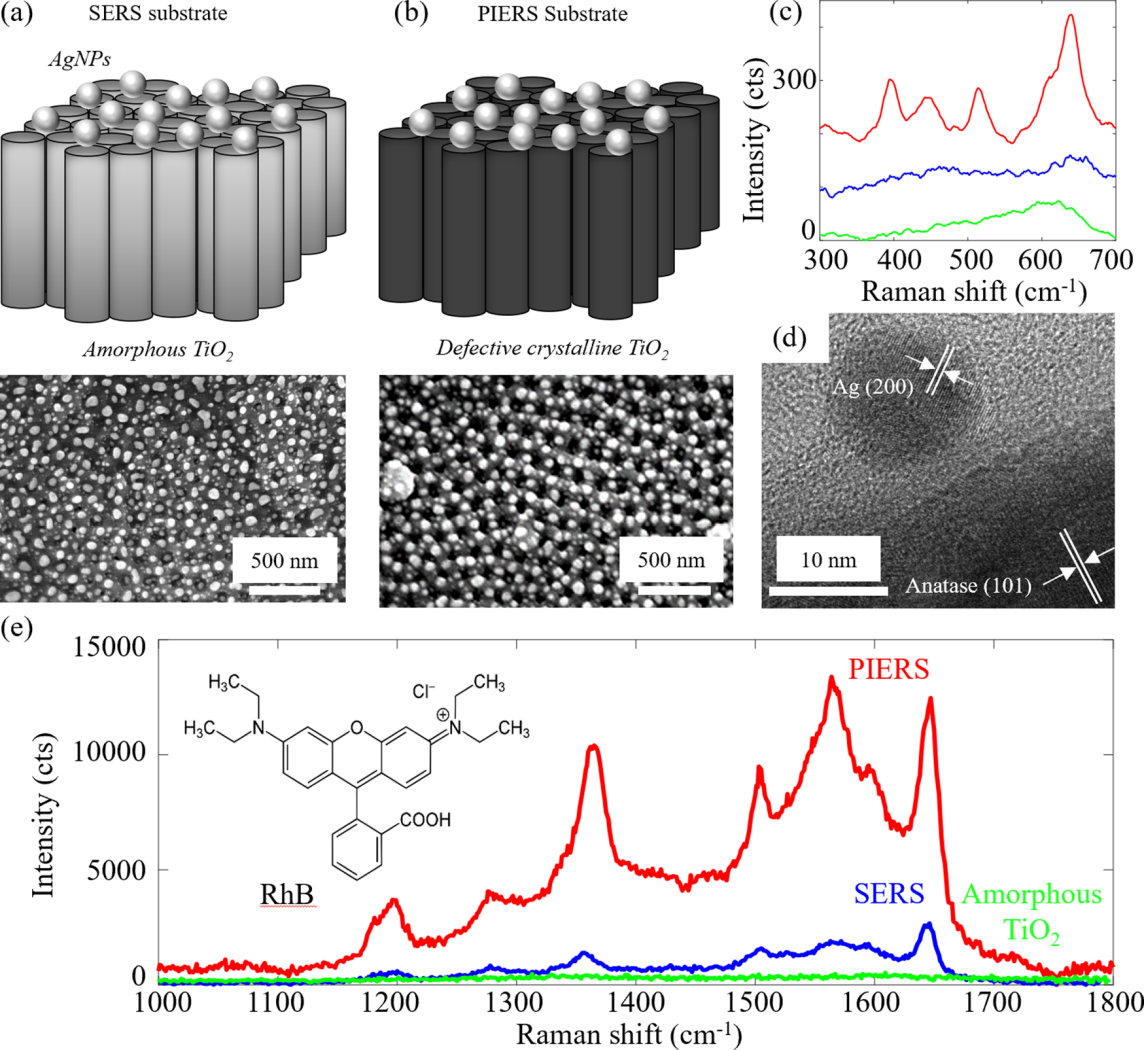
Enhanced Raman in PIERS substrates. (a) SEM image of SERS (Ag nanoparticles, AgNPs on amorphous TiO_2_) substrate and (b) PIERS (AgNPs on crystalline defective TiO_2_, annealed at 600 °C for 1 h in Ar prior to AgNP formation) substrate with schematics above. (c) Raman spectra of bare as-anodized TiO_2_ substrate (green), SERS substrate (blue), and PIERS substrate (red) with crystalline anatase (395 (B_1g_), 515 (A_1g_), and 639 (E_g_) cm^–1^ ^16^) and rutile peaks (446 (E_g_) and 606 (A_1g_) cm^–1^). (d) TEM image of PIERS substrate with lattice fringes and phase indicated (detail in Figure S7). (e) Raman spectrum of Rhodamine B (10 μM) on a bare TiO_2_ substrate, a SERS substrate (enhancement factor 3.4 × 10^5^), and a PIERS substrate (enhancement factor 2.3 × 10^6^) averaged over 10 locations on a sample.

The PIERS signal of adsorbed Rhodamine B (RhB) displays close to an order-of-magnitude greater enhancement factor (Table 1) over the equivalent SERS signal (Figure 1e).^17^ The enhancement factors listed in Table 1 are averaged over the surface of the substrate and calculated using several different peaks (detailed calculations in Sections I and II of Supporting Information). In Figure S6, we demonstrate the effect of the AgNP size distribution on Raman signal enhancement by tuning the Ag dewetting time to sweep its surface plasmon into resonance with the 488 nm Raman excitation laser. An order-of-magnitude enhancement in PIERS relative to SERS is always observed, despite changing the particle size distribution, indicating this result is robust with respect to the polydispersity of the AgNPs.

**Table 1:** Enhancement Factors for SERS vs PIERS

		enhancement factor		
	RhB [M]	1648 cm^–1^	1358 cm^–1^	average
PIERS	10^–5^	1.6 × 10^6^	2.9 × 10^6^	2.3 × 10^6^
SERS	10^–5^	3.1 × 10^5^	3.7 × 10^5^	3.4 × 10^5^

We map out the key steps (Figure 2) in the flow of electrons induced by the excitation laser to reveal the PIERS enhancement and its difference to conventional SERS.

**Figure 2:**
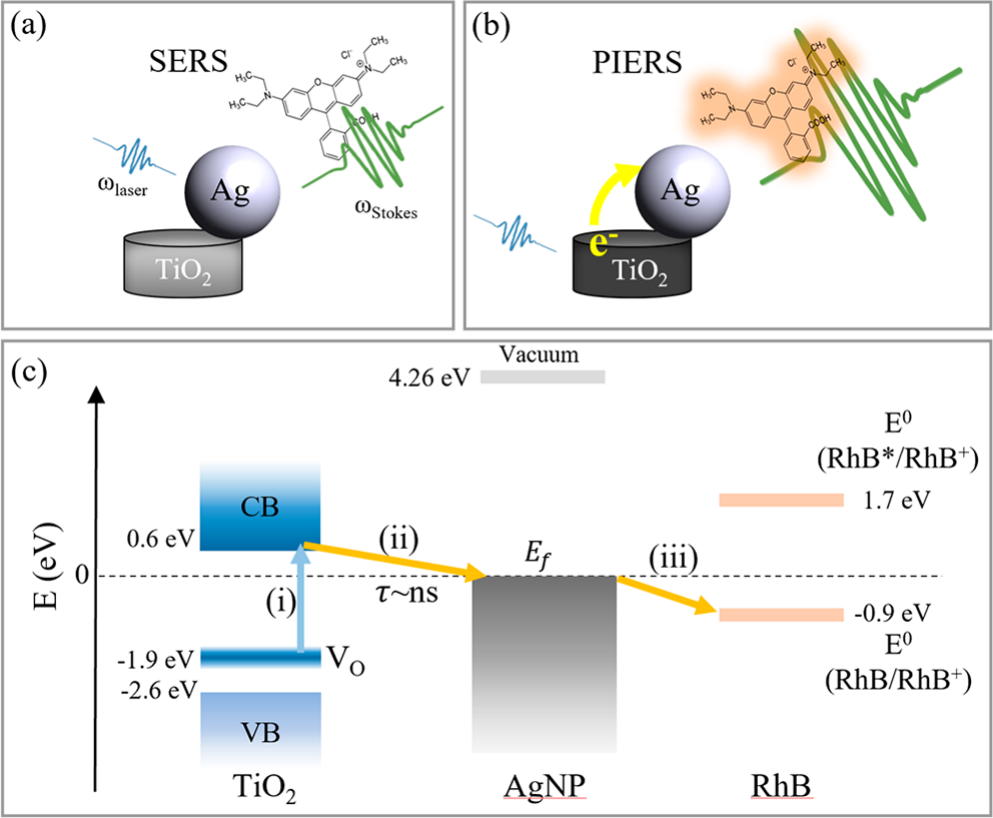
SERS vs PIERS. (a) Plasmonic field enhancement mechanism for SERS. (b) Charge transfer enhancement for PIERS. (c) PIERS mechanism: (i) excitation of electrons from defect states to the conduction band, (ii) electron transfer from TiO_2_ to AgNPs Fermi level (*E*_f_) with a ns decay time (τ), and (iii) electromagnetic enhancement and charge transfer interaction of AgNPs with the RhB probe molecule.

*(i) Excitation of Electrons in TiO_2_ Defect States to the Conduction Band.* The first step (Figure 2c(i)) involves the absorption of visible (488 nm wavelength, 2.54 eV) photons from the Raman excitation laser by oxygen vacancy defect states within the crystalline TiO_2_ PIERS substrate. This process is not permitted in amorphous TiO_2_, which has a large electronic bandgap.^41,43−45^ Annealing amorphous TiO_2_ at 600 °C in argon transforms it to defective crystalline TiO_2_ (Figure 1c) which has an increased absorbance at wavelengths of >400 nm (Figure 3a and Figure S11).^41^ The energy level of the oxygen vacancy states is 0.7 eV above the valence band of TiO_2_ (Tauc plot, Figure S12). The Ti 2p XPS spectrum (Figure 3b) of the PIERS substrate also confirms the presence of Ti^3+^ peaks (457 and 463 eV), associated with oxygen vacancies in addition to the Ti^4+^ peaks (459 and 464 eV).^46−48^ After the deposition and thermal dewetting of AgNPs, plasmon resonances are visible at 443 and 400 nm in the PIERS and SERS substrates (Figure 3a). The broad plasmon peaks are nearly resonant with the incident 488 nm Raman laser.

**Figure 3:**
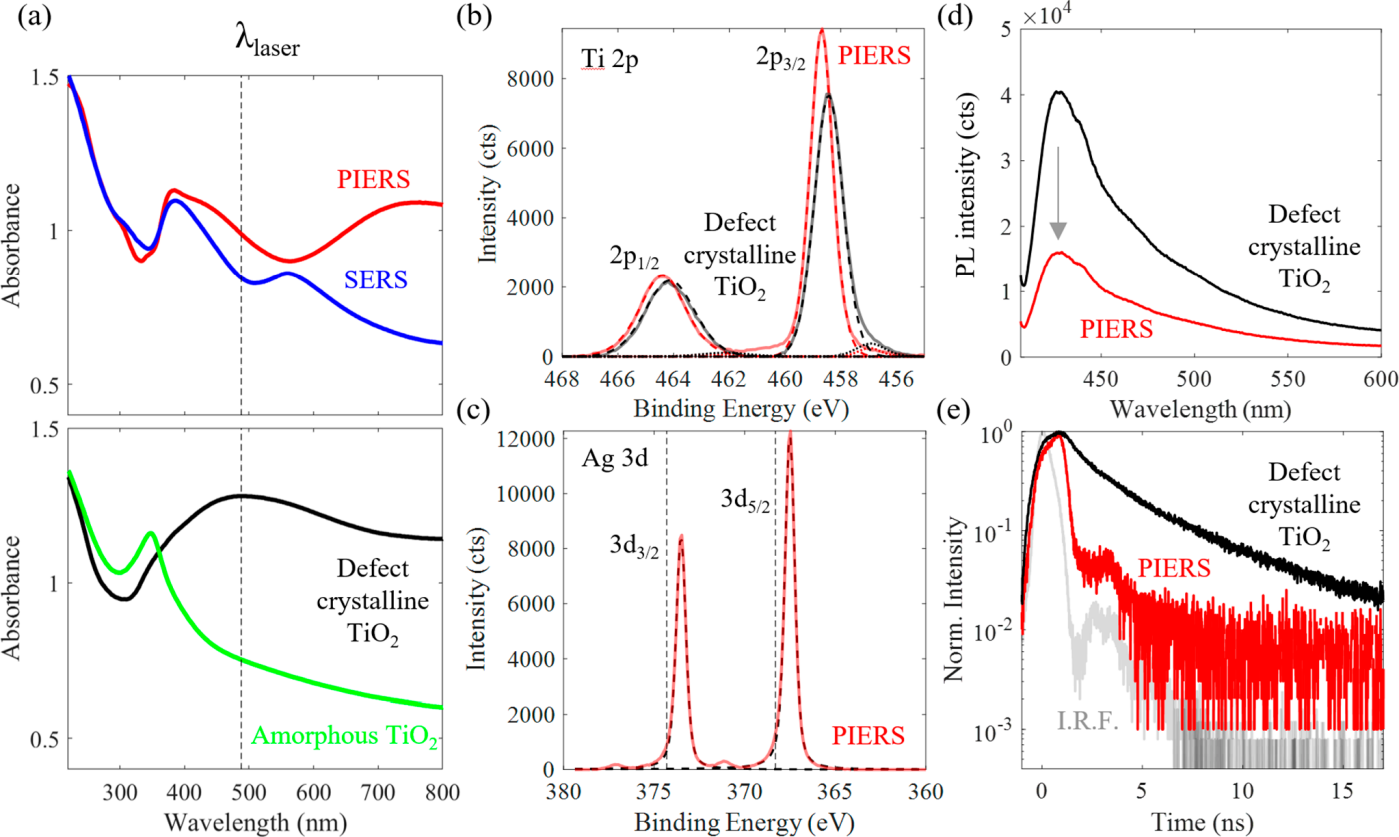
Charge transfer from TiO_2_ oxygen vacancies to AgNPs. (a) Diffuse reflectance UV–vis spectra of (top) PIERS and SERS substrates; (bottom) defect crystalline TiO_2_ (annealed at 600 °C in Ar for 1 h) and amorphous TiO_2_. The gray dotted line indicates the center wavelength of the Raman excitation laser (488 nm). (b) Ti 2p XPS of the PIERS and SERS substrates, with dotted lines indicating mixed Gaussian–Lorentzian fits to the 2p peaks for Ti^4+^ and Ti^3+^. (c) Ag 3d XPS of PIERS substrate, where the vertical dotted lines are the standard binding energies of metallic Ag ^56^ and dotted lines indicating mixed Gaussian–Lorentzian fits to the 3d peaks. (d) Photoluminescence spectra of the PIERS substrate and defect crystalline TiO_2_ (325 nm excitation laser, 1 mW). (e) Fluorescence decay lifetime at 460 nm of the PIERS substrate, defect crystalline TiO_2_, and instrument response function, I.R.F.

Strong absorption of light by defect states is a necessary but not sufficient condition to facilitate the charge transfer required for PIERS enhancement. SERS and PIERS substrates both have defect states due to the presence of carbon and fluorine impurities (Figure S9),^49^ in addition to oxygen vacancy defect states. Hence, both show a reduced onset of band absorption (SERS, 2.2 eV; PIERS, 2.4 eV; Figure S12) compared to pure anatase TiO_2_ (3.2 eV). However, only the crystalline PIERS substrate allows electrons excited into the conduction band to migrate into the nearest AgNP. SERS substrates made of amorphous TiO_2_ have higher nonradiative recombination rates and reduced electron mobility, which decreases the density of photoexcited electrons undergoing charge transfer into AgNPs.

*(ii) Electron Transfer from TiO_2_ to Ag.* Photoinduced electron transfer from TiO_2_ to AgNPs on the PIERS substrate was measured by the suppression of photoluminescence (PL) in the PIERS substrate (with AgNPs) compared to the defective crystalline TiO_2_ substrate (without AgNPs) in Figure 3d and Figure S16. The PL peaks, obtained by excitation with a 325 nm laser, are assigned to self-trapped excitons (516 nm),^50^ hydroxyl surface states (463 nm),^51^ and oxygen vacancy states (546 nm).^52^ The PL suppression is due to the reduced radiative recombination of electrons in crystalline TiO_2_ due to electron transfer to AgNPs, with reverse-transfer prohibited by the Schottky barrier formed between Ag and TiO_2_.^53,54^ In addition to the photoinduced charge transfer, there is also strong electrostatic Ag–TiO_2_ interfacial charge transfer resulting in a blueshift in the Ti 2p XPS spectrum and a corresponding redshift in the Ag 3d spectrum.^47^ The Ti^4+^ 2p_3/2_ peak binding energy increases from 458.4 eV in the defective crystalline TiO_2_ substrate to 458.7 eV in the PIERS substrate (Figure 3b). The Ag 3d_5/2_ spectrum correspondingly decreases for the PIERS substrate (367.5 eV), compared to bulk Ag (368.3 eV) (Figure 3d). The binding energies indicate that the AgNPs are not oxidized and remain in the Ag(0) metallic state.^55^

Time-resolved PL spectra show that there is an additional PL peak at 675–700 nm after 12 ns for all PIERS and SERS samples (Figure S17a–d), which is attributed to a carrier detrapping process from the defective sub-bandgap states. These additional PL peaks can be found in all samples, indicating that these defect states are due to the insufficient supply of oxygen inside the electrolyte during anodization.^41^ Time-correlated single photon counting (TCSPC) (Figure 3e and Figure S18) show that the carrier lifetime probed at 460 nm is significantly shorter when AgNPs are deposited on the surface (PIERS) of the defective crystalline TiO_2_ substrate, indicating efficient charge transfer to AgNPs or a Purcell-effect shortened decay lifetime.^1^ The nanosecond lifetime and efficient charge transfer thus facilitate the final step of the process, which is the photoinduced charge transfer to the adsorbed RhB molecules. Excitation wavelength dependent Raman spectra confirm the impact of charge transfer on the Raman enhancement (Figure S19), with the sub-bandgap 785 nm excitation showing no PIERS enhancement. The higher the laser excitation energy, the larger is the PIERS enhancement (488 nm > 532 nm > 785 nm) as more photocarriers are generated and transferred from TiO_2_ to AgNPs. Modifying the substrate’s crystallinity through different annealing temperatures also reveals an optimum in PIERS enhancement. This optimum is due to an increasing rutile fraction which has a lower charge mobility and decreased defect-assisted light absorption than anatase, further supporting this hypothesis (Supporting Information Section VIII).

*(iii) Electromagnetic and Charge Transfer Interaction between AgNPs and RhB.* As we have established a mechanism for charge transfer of photoexcited electrons from TiO_2_ to AgNPs, there now remain several possibilities for the final step. Below, we address each hypothesis and conclude that the strong adsorption of RhB to Ag increases RhB’s intrinsic Raman polarizability, which is further enhanced by photoinduced charge transfer from TiO_2_ to Ag. Charge transfer from AgNPs to RhB is possible as the reduction potential *E*^0^(RhB/RhB^+^)^57−59^ lies below the Fermi level of AgNPs (Figure 2). The alternative processes considered are

1. *Increased electron density on AgNPs increases the electromagnetic enhancement*: As more electrons are transferred to AgNPs, the electromagnetic field around the particles is enhanced, which can create stronger Raman signals. However, we can discount the purely electromagnetic contribution to the PIERS enhancement due to increased electron density, as this is not predicted to increase the enhancement factor by more than 13%.^60−63^
2. *Suppression of the fluorescence background of RhB due to interaction with AgNPs or photoexcited TiO*_*2*_: Raman spectra collected in Figure S3a show an increase rather than a decrease in the background fluorescence, consistent with the literature.^64^ We were also unable to measure any Raman enhancement of RhB on bare amorphous or defective TiO_2_. Hence, the fluorescence quenching mechanism can be discounted.
3. *Metal-to-ligand-charge-transfer (MLCT) resonance Raman enhancement*: A third possibility, which is favored by current studies,^19,25,29,32,36,37^ is ligand-to-metal charge transfer (LMCT) or metal-to-ligand charge transfer (MLCT) bands forming between Ag and RhB, which adds a further resonance Raman enhancement^1^ in addition to the plasmonic field enhancement. Unfortunately, it is not possible to optically measure the spectrum of the charge transfer band, as it would be obscured by the plasmonic resonance. Hence, the energy level diagram in Figure 2 was rigorously constructed (Supporting Information Section VI) using a range of spectroscopic/electrochemical techniques (XPS, UV–vis, cyclic voltammetry). Using cyclic voltammetry (Figure 4 and Figure S15), the band alignment of RhB was determined from its redox potentials. From the onset redox potential of *E*_ox_^0^ = 0.89 V vs Ag/AgCl (details in Supporting Information Section VI), the HOMO level can be found by converting to the potential relative to the vacuum: *E*_HOMO_ = −(*E*_ox_^0^ + 4.28) = −5.2 eV from the vacuum. The measured HOMO level is consistent with previous measurements.^57,59,65^ From the experimentally determined band diagram, there are no transitions possible from any of the occupied energy levels (oxygen vacancy state, valence band of TiO_2_, Fermi level of AgNPs, and HOMO of RhB) to unoccupied levels (conduction band of TiO_2_ and LUMO of RhB) with a photon at 488 nm (2.54 eV), hence excluding a conventional LMCT/MLCT resonance Raman enhancement.
4. *Photoinduced electron transfer to RhB increasing the Raman polarizability:* The final explanation for PIERS enhancement is a change in the intrinsic Raman cross-section of the chemisorbed molecule on the AgNP surface vs the cross-section of a nonchemisorbed molecule.^4^ The binding energies of three different adsorption geometries were calculated: Ag bonding via the amine functional group (RhB–amine–Ag), the stacking of the xanthene ring (RhB–xanthene–Ag), or the carboxyl group (RhB–carboxyl–Ag).^66−68^ The binding via the carboxyl group is very unfavorable (Δ*E*_ads_ = +13 eV), while the binding via the xanthene and amine groups are very close in terms of energetic stability (Δ*E*_ads_ = −2 eV).

**Figure 4:**
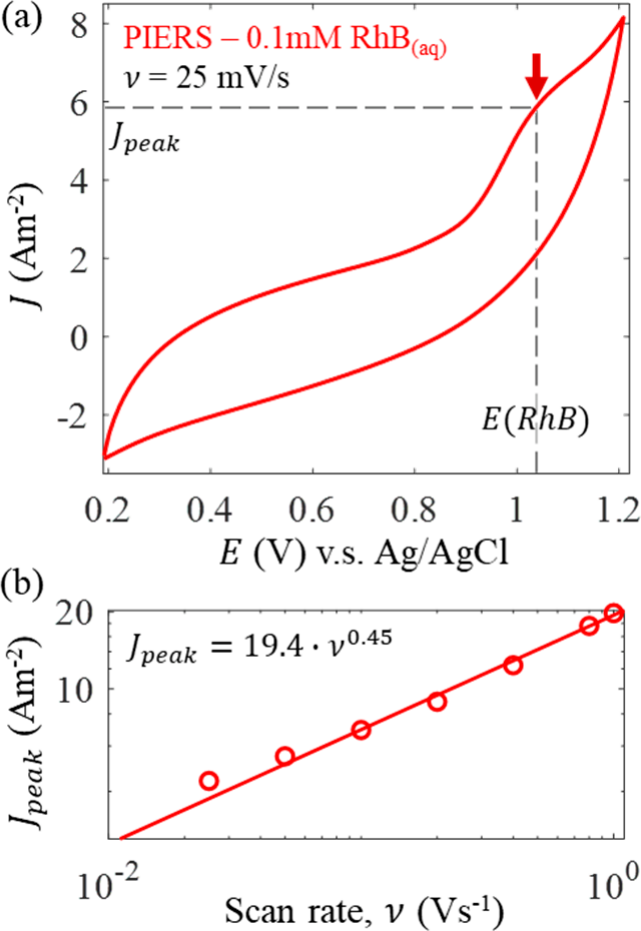
RhB energy alignment and charge-transfer on PIERS substrate. (a) Cyclic voltammogram of PIERS substrate at a scan speed ν of 25 mV/s in a 0.1 mM nitrogen-sparged solution of aqueous RhB. Redox peak of RhB is indicated with an arrow along with the redox potential E(RhB) and peak current *J*_peak_. (b) Peak current *J*_peak_ vs scan rate ν indicating efficient diffusion-limited charge transfer from the PIERS substrate to RhB.

The most likely binding geometry was determined by calculating the Raman modes of RhB+ using DFT in Figure 5a (B3PW91/6-31G+(d,p) and LANL2DZ(for Ag)) and applying Moscovitz’s SERS surface selection rules:^69^ normal modes with a large polarizability component normal to the metal’s surface will be enhanced. In Figure 5a, both the PIERS and SERS substrates show an enhanced Raman intensity of the antisymmetric xanthene ring stretch at ∼1370 cm^–1^ (DFT, 1344 cm^–1^) and symmetric amine stretches at ∼1200 cm^–1^ (DFT, 1260 cm^–1^) relative to the pure xanthene ring stretch at 1646 cm^–1^. As the enhanced xanthene ring stretches have their displacement vectors aligned along the long axis of the RhB molecule (Figure 5c), this implies that the binding needs to occur via a functional group face that is aligned to RhB’s long axis. This leaves only one option: the nitrogen fragment of the RhB must bind strongly to the Ag surface. This conclusion is supported by the increased intensity of peaks associated with a xanthene ring carbon–nitrogen stretch (1380–1390 cm^–1^). If RhB were lying fully flat, it would be the out-of-plane modes that would be enhanced, at the expense of the in-plane modes, which is not observed. Marchi et al.^64^ also found that RhB prefers to coordinate to Ag via its amine groups. As the amine’s motion is hindered, the fluorescence quantum yield should increase as the greater rigidity reduces the rate of internal conversion, which we observe as an overall increase in the Raman background (Figure S3). However, the absolute determination of the range of likely binding geometries is challenging and dependent on nuances such as local pH. Despite that, all simulations with Ag present increase the Raman polarizability of RhB except for coordination to the xanthene, which further supports the coordination of Ag to N dominating the SERS and PIERS spectra.

**Figure 5:**
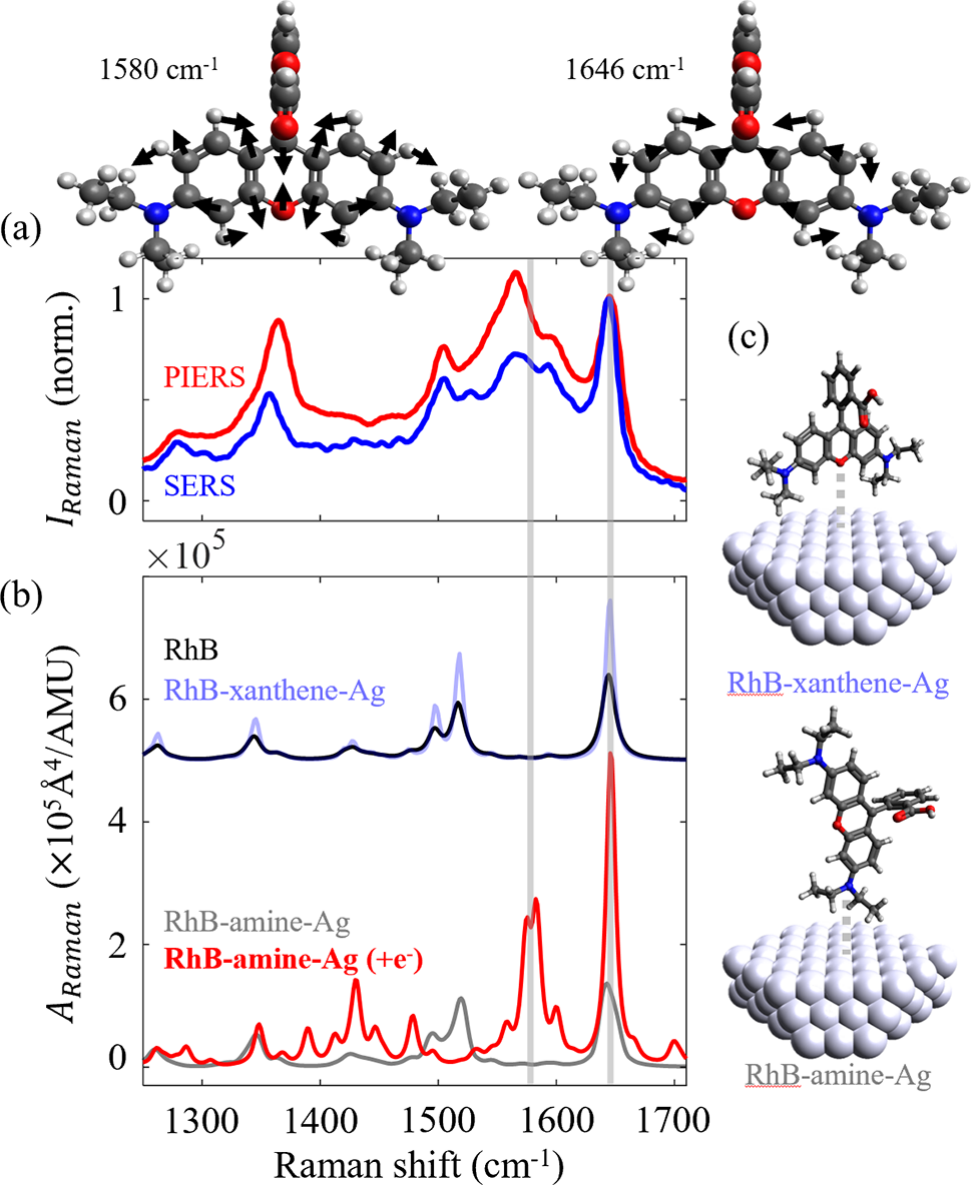
Charge transfer from AgNPs to RhB molecules enhances Raman polarizability. (a) Top: scaled Raman spectra of PIERS, SERS, and normal Raman spectra of RhB (scaled to the peak intensity at 1647 cm^–1^). (Top) Raman-active vibrational modes of RhB cation calculated via DFT. Arrows indicate the magnitude and direction of the displacement vectors for each vibration. (b) Dynamic Raman activities (*A*_Raman_) predicted via DFT (B3PW91/6-31G(d,p)+ LANL2DZ, 488 nm excitation) for different binding geometries of RhB on Ag and unbound RhB. Raman transition frequencies were scaled by 0.963. (c) Associated binding geometries of RhB on Ag (bound via xanthene ring’s oxygen and via amine group).

The impact of charge transfer was modeled by calculating the dynamic Raman scattering activity at an excitation wavelength of 488 nm for the most likely binding geometry (RhB^+^–amine–Ag, Figure 5b). The RhB–amine–Ag spectrum has a very similar spectrum and scattering intensity to the bare RhB. Upon electron donation to form the reduced RhB–amine–Ag (+1e^–^) complex, the overall predicted Raman scattering intensity increases, and the band at 1580 cm^–1^ is now enhanced. The combination of the RhB–amine–Ag and reduced RhB^+^–amine–Ag (+1e^–^) DFT-predicted spectra now matches the experimental SERS and PIERS spectra very well, and their relative intensities indicate efficient charge transfer. Raman experiments performed on 1-octanethiol do not show a PIERS enhancement relative to SERS, due to misaligned energy levels for charge transfer, with a deep HOMO level of −3.5 eV ^70^ from a metal’s Fermi level and a HOMO–LUMO gap of 8–9 eV,^71^ which supports this mechanism. Furthermore, based on the square root dependence of peak current to scan rate in Figure 4b, electron transfer to RhB is efficient and diffusion-limited, indicating that photoinduced electron transfer can readily occur.

We elucidate the mechanism of photoinduced enhanced Raman spectroscopy (PIERS) on heterostructures of TiO_2_ nanotubes and AgNPs. PIERS substrates show a 680% enhancement of Raman signals compared to the equivalent SERS substrates. Using a range of spectroscopic techniques, we show the key steps of the PIERS mechanism: (i) absorption of visible light by defect states in TiO_2_, (ii) population of the conduction band of TiO_2_, (iii) electron transfer to the Fermi level of AgNPs, and (iv) electron transfer from AgNPs to the adsorbed RhB molecule. DFT simulations suggest that the final step increases the intrinsic polarizability of the molecule. Contrary to previous reports,^17,26^ we find no evidence for charge transfer resonance Raman enhancement due to photon-assisted electron transfer from the substrate to Rhodamine dyes. We also highlight the balance that needs to be struck between defects that increase Raman laser absorption and crystallinity that increases charge mobility and transfer to AgNPs.

By identifying the elementary steps of the PIERS enhancement process, the design rules for fabricating a PIERS substrate are clarified: A PIERS substrate requires a crystalline semiconductor capable of absorbing the visible Raman excitation laser (via sub-bandgap defect states or an appropriate bandgap), a semiconductor conduction band energy greater than the Fermi level of plasmonic nanoparticles, and a probe molecule capable of undergoing charge transfer and whose Raman polarizability is enhanced and not suppressed upon charge transfer. These detailed requirements establish a design protocol for PIERS, identify which analytes will benefit from PIERS enhancements, and will have broad impact in the field of enhanced Raman spectroscopies.^72,73^ This work provides key insight into the PIERS mechanism, which will impact the use of SERS to characterize metal–semiconductor interfaces for catalysis^74^ and sensing.^18,75,76^

## Experimental Section

*Materials and Methods. Ti Foil Preparation.* Titanium foil (>99.6% Ti) was cut into sheets of 40 mm × 20 mm × 0.5 mm, mechanically polished, and ultrasonically cleaned in acetone (99% anhydrous), ethanol (99% anhydrous), and deionized water. Prior to anodization, the foil samples were stored in ethanol to prevent tarnishing.

*Double Anodization of TiO_2_.* The electrolyte used for anodization contained 0.25 wt % ammonia fluoride (Sigma-Aldrich, 98% purity), ethylene glycol (ECP-Laboratory Reagent, 99% anhydrous), and 2 vol % deionized water. The first anodization was performed at 60 V for 25 h. The 25 h anodized specimens were then immersed in water for 10 min and dried in air. The oxide layer was subsequently peeled off, and the Ti foil was cleaned in an ultrasonic bath and dried in air. The second anodization was performed using the same aged electrolyte at 80 V for 5 min. After the second anodization, the double anodized TiO_2_ was immersed into ethanol solution for 24 h to prevent cracking during further annealing process.

*Annealing and Thermal Dewetting.* The annealing process was performed in an argon atmosphere furnace at 600 °C for 1 h to crystallize the amorphous double anodized TiO_2_ and introduce oxygen vacancy states. A magnetron sputter (NANO 36, Kurt J. Lesker Company) was then used to deposit Ag layer. The deposition time was varied for 10, 20, and 40 s with the power of 100 W in a 3 mTorr Ar chamber. The sputtered substrates were subsequently thermally treated at 250 °C for 1 h in the Ar furnace and cooled down slowly to room temperature in the furnace.

*Characterization. Raman Spectroscopy.* Raman spectra were acquired using a Renishaw RM1000 Raman microprobe comprising a single grating spectrograph of 2400 g/mm with a holographic notch filter removing Rayleigh scattered light below 150 cm^–1^, with a 20× objective lens (N.A. of 0.5) providing a spot size at the surface of 1–2 μm and entrance slit width of 50 μm. Excitation at 488 nm was provided by an air-cooled Spectra-Physics argon ion laser at 1 mW. The test molecule used in this study was Rhodamine B (RhB). The acquisition time was 60 s per spectrum with three accumulations. For tests on the PIERS and SERS substrates, ∼5 μL of RhB solution (10^–5^ mol L^–1^, 10^–6^ mol L^–1^, and 10^–7^ mol L^–1^) was deposited onto each substrate and dried in the air for 5 min prior to Raman spectra acquisition. All spectra presented are collected over at least three locations and averaged.

*Scanning electron microscopy* (Philips XL 30, 5 kV) was used to analyze the surface morphology and particle size distribution for the PIERS substrates with different Ag deposition time. *High-resolution transmission electron microscopy* (Tecnai FEG20) was used to examine the crystalline structure of the PIERS substrates.

*X-ray photoelectron spectroscopy* (Kratos Axis ultra DLD) using a monochromatic Al Kα X-ray source of 100 W was performed to investigate the chemical composition and defect nature of the TiO_2_ substrates. The XPS chamber pressure was about 10^–9^ Torr, and core level scans were carried out at a pass energy of 20 eV. The spectrum was fitted using a Shirley background and with Gauss–Lorentz peaks.

*UV–vis spectroscopy* (Shimadzu UV-2600 spectrophotometer fitted with an integrating sphere attachment) was performed over the wavelength range 220–1400 nm, with BaSO_4_ powder as a reference.

*Photoluminescence Spectra.* Steady-state and time-resolved PL spectra were recorded by a gated intensified CCD camera (Andor Star DH740 CCI-010) connected to a grating spectrometer (Andor SR303i). The pulsed output from a mode-locked Ti:sapphire optical amplifier (Spectra-Physics Solstice, 1.55 eV photon energy, 80 fs pulse width, 1 kHz repetition rate) was used to produce 400 nm excitation via second harmonic generation in a β-barium borate crystal. The iCCD gate (width 5 ns) was electronically stepped in 5 ns increments, relative to the pump pulse, to enable ns-temporal resolution of the PL decay.

*Time-correlated single photon counting* decay curves were obtained by a Pico Quant LDH407 laser diode at 407 nm with a repetition rate of 40 MHz. The emission signal was selected with a monochromator to obtain the desired emission wavelength and detected by a Hamamastu R3809U-50 photomultiplier detector. Long-pass filters were utilized to remove the scattered photons from the excitation laser. The decay curve is recorded for the emission at 460 nm.

*Electrochemical measurements* were performed using a three-necked glass flask, with a Pt mesh counter-electrode (Alfa Aesar) and saturated Ag/AgCl reference electrode (3 M KCl), controlled via a Autolab PGSTAT204 (Metrohm) potentiostat. Au electrodes were fabricated by thermal evaporation of 5 nm Cr, and 100 nm layer of Au onto a Si wafer which was subsequently diced to shape with copper wires attached. The PIERS substrate was converted to an electrode by blocking the Ti back surface with insulating tape and electrically contacting the exposed Ti substrate.

*Computational Methods. Density functional theory* calculations of the predicted vibrational frequencies of bare RhB cation and RhB chemically adsorbed onto Ag atoms were peformed in Gaussian 09W, using the B3PW91 exchange–correlation functional and an effective core-potential model for the metal center. The main group elements (C, H, P, S, etc.) were modeled by the triple-ζ basis set 6-31G(d,p)+. The Ag center basis set was approximated with the LANL2DZ pseudopotential. All geometry optimizations were performed with a gradient descent search technique after molecular dynamics optimization, and stationary points were found via frequency analysis. All optimized geometries have positive frequencies to indicate a real structure. The Raman spectra were calculated both within the static limit and dynamic limit with frequency-dependent polarizabilities calculated at an excitation wavelength of 488 nm. Different charge states of the RhB–Ag complex were performed using the neutral spin singlet and a singly positively charged spin doublet.

## Supporting Information Available

Section I, Raman spectra and substrate synthesis; Section II, nanoparticle size distribution characterization and Raman spectra; Section III, chemical characterization of PIERS and SERS substrates (XPS, TEM, Raman, XRD); Section IV, optical characterization of PIERS and SERS substrates; Section V, Raman peak fitting and analysis; Section VI, band alignment of semiconductor-metal-molecule; and Section VII, time-resolved photoluminescence (PDF)
Transparent Peer Review report available (PDF)

## Abbreviations

SERS: surface-enhanced Raman spectroscopy
PIERS: photoinduced enhanced Raman spectroscopy
