# Supplementary material for: Understanding the Chemical Mechanism behind Photoinduced Enhanced Raman Spectroscopy
Source: J Phys Chem Lett. 2023 May 11;14(19):4607–16. doi: 10.1021/acs.jpclett.3c00478 (PMC10201573; doi:10.1021/acs.jpclett.3c00478)
Supplement: Supplementary file 1 — jz3c00478_si_001.pdf [file jz3c00478_si_001.pdf]

Supplementary Information

Understanding the Chemical Mechanism Behind Photo-Induced Enhanced Raman Spectroscopy

Junzhi Ye‡[1,2,3], Rakesh Arul‡*[1,2,3,4,5,6,7], Michel K. Nieuwoudt[1,4,5,6], Junzhe Dong[2], Ting Zhang[2], Linjie Dai[3], Neil C. Greenham[3], Akshay Rao[3], Robert L. Z. Hoye[8], Wei Gao*[2], M. Cather Simpson*[1,2,4,5,7]

1. The Photon Factory, the University of Auckland, Auckland, New Zealand
2. Department of Chemical and Materials Engineering, the University of Auckland, Auckland, New Zealand
3. Cavendish Laboratory, University of Cambridge, JJ Thomson Avenue, Cambridge, United Kingdom
4. The MacDiarmid Institute for Advanced Materials and Nanotechnology, New Zealand
5. The Dodd Walls Centre for Quantum and Photonic Technologies, New Zealand
6. School of Chemical Sciences, the University of Auckland, Auckland, New Zealand
7. Department of Physics, the University of Auckland, Auckland, New Zealand
8. Inorganic Chemistry Laboratory, University of Oxford, South Parks Road, Oxford, United Kingdom

Section I: Raman spectra and substrate synthesis

Section II: Nanoparticle size distribution characterization and Raman spectra

Section III: Chemical characterization of PIERS and SERS substrates (XPS, TEM, Raman, XRD)

Section IV: Optical characterization of PIERS and SERS substrates

Section V: Raman peak fitting and analysis

Section VI: Band alignment of semiconductor-metal-molecule and electrochemical characterization

Section VII: Time-Resolved Photoluminescence

Section VIII: Effect of annealing temperature on PIERS effect

Section IX: Effect of non-charge transfer compatible molecules on PIERS effect

Section I: Raman spectra and substrate synthesis

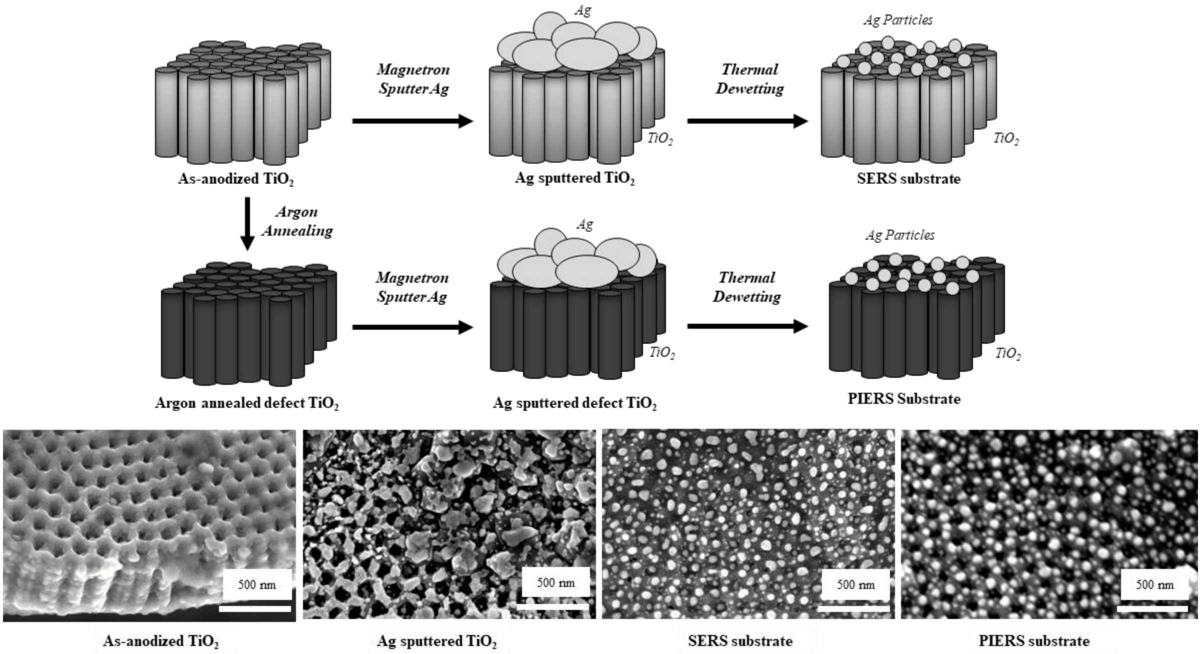

Figure S1 Synthesis procedure for SERS and PIERS substrate, with corresponding SEM images below

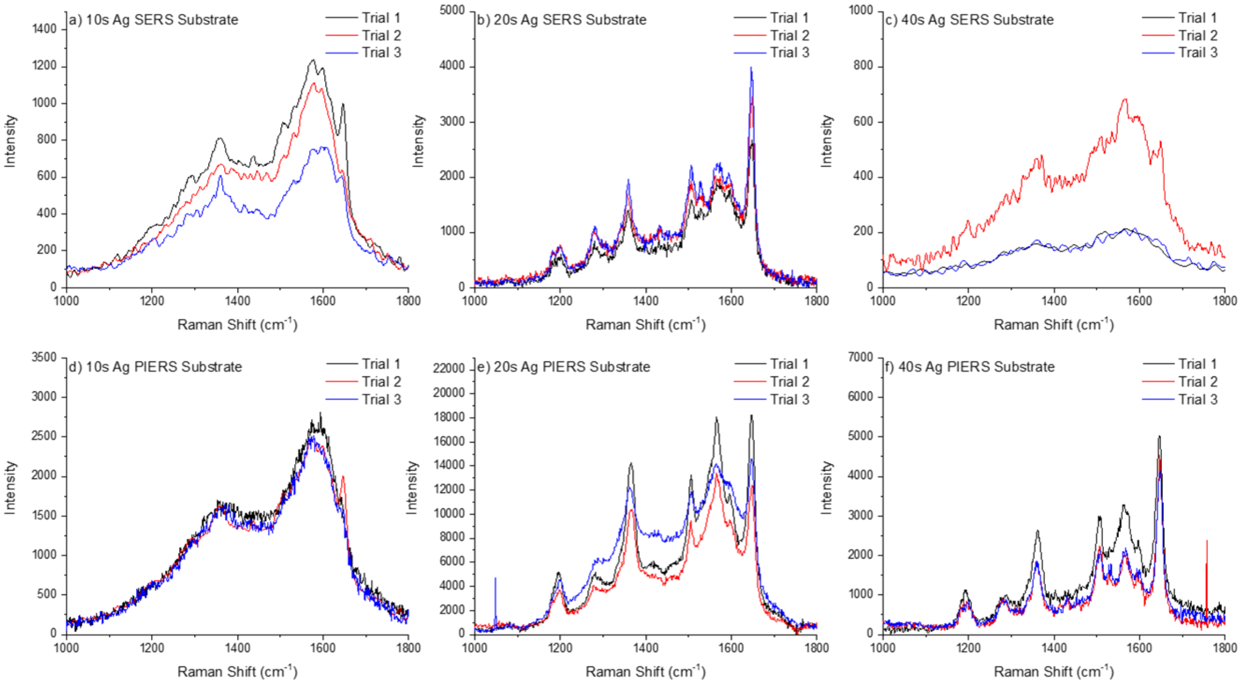

Figure S2: Reproducibility Study. a)-c) Enhanced Raman spectra of RhB (10^{-6}M) on SERS samples with different Ag sputtering time, 10s, 20s and 40s, respectively. d)-e) Enhanced Raman spectra of RhB (10^{-6}M) on PIERS samples with different Ag sputtering time, 10s, 20s and 40s, respectively.

The Raman enhancement factor (EF) is calculated using Eqn (S1) below, via integrating the area of the peak beneath two different Raman bands (1648 cm^{-1} and 1358 cm^{-1}) to give I_{SERS} and I_{bulk}. The intensities are then scaled by the molar concentration of the RhB analyte in the case of the bulk powder v.s. the molecule of a certain concentration deposited on the SERS/PIERS substrates, which is a well-established method to estimate the EF[1, 2]. This EF is an underestimate as the likely concentration of the molecule is smaller than that shown as some molecules will not remain bound on the surface or within SERS hotspots.

$$\mathrm{EF} = \left(\frac{I_{SERS}/N_{SERS}}{I_{bulk}/N_{bulk}}\right) = \left(\frac{I_{SERS}/I_{bulk}}{C_{SERS}/C_{bulk}}\right) \qquad \text{Eqn. (S1)}$$

Table S1: Calculation of the Enhancement Factor (EF)

Substrate	***Conc. RhB [M]***	***Peak area (cts.cm/mW.s)***		***EF***		
		1648 cm^{-1}	*1358 cm^{-1}*	*1648 cm^{-1}*	*1358 cm^{-1}*	*Average*
PIERS	10^{-5}	381548	492984	1.6×10^{6}	2.9×10^{6}	2.3×10^{6}
SERS	10^{-5}	73040	64171	3.1×10^{5}	3.7×10^{5}	3.4×10^{5}
Raman	2.75	64219	47272	1	1	1

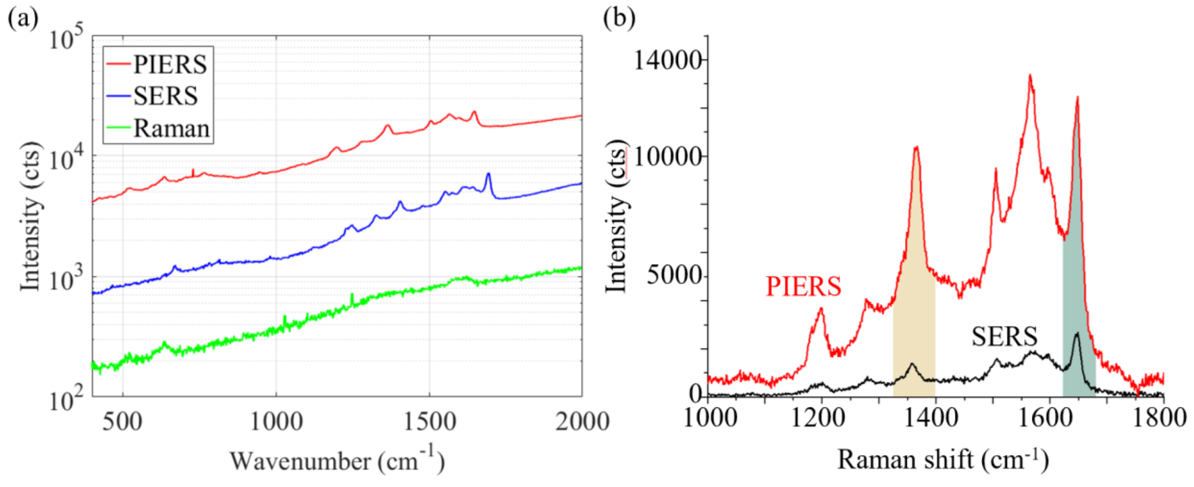

Figure S3 Raman spectra from powder, SERS, and PIERS substrates. (a) Non background corrected Raman spectra of RhB using PIERS, SERS, and no enhancement on aluminium foil (all at 10-5 M concentration of RhB) showing the increase in the fluorescence background. (b) Shaded integrated area taken for EF calculations centered at 1358 and 1648 cm^{-1}.

Section II: Nanoparticle size distribution characterization and Raman spectra

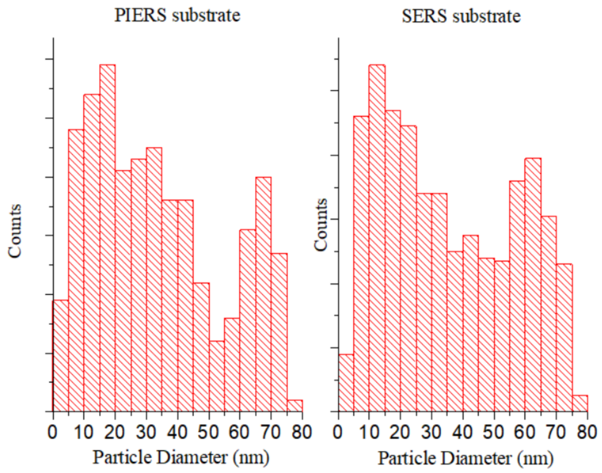

Figure S4 Ag Particle Distribution on PIERS vs. SERS substrate

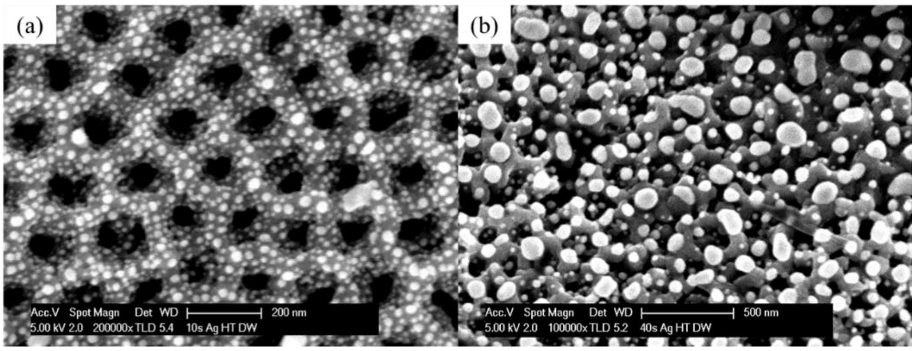

Figure S5 PIERS substrates with off-resonant Ag nanoparticles (a) 10s Ag deposition, (b) 40s Ag deposition, at same pressure and other magnetron sputter settings as the on-resonant PIERS substrate.

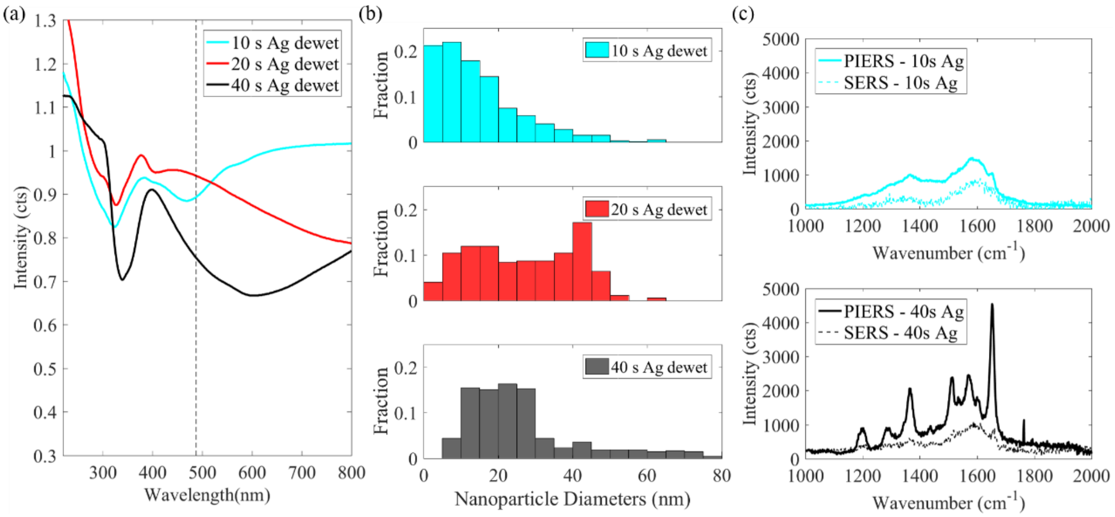

Figure S6 PIERS v.s. SERS enhancement for different AgNP size distributions: AgNPs size distribution tuning on PIERS substrates - via varying the sputtering time of Ag to 10 s, 20 s and 40 s prior to de-wetting. (a) UV-Vis absorption spectrum of PIERS substrates. (b) Nanoparticle diameter histogram obtained from SEM measurements for Ag sputtering times of 10 s, 20 s, and 40 s and subsequent dewetting. PIERS v.s. SERS Raman spectra averaged over three locations for (c) 10 s Ag sputtering time and (d) 40 s Ag sputtering

time.

The PIERS substrate enhancement dropped to 10^5 due to the detuned surface plasmon resonance of Ag for the 10 s and 40 s sputtered substrates. For both 10 s and 40 s dewetting, we see quantitatively different nanoparticle size distributions compared to the 20 s model PIERS substrate, yet despite that we observe a higher Raman signal for the PIERS substrate than the SERS substrate. The constant in this experiment is the presence of the same defective crystalline TiO_2 in all PIERS substrates, and the absence of crystallinity in the SERS substrates. This lends further evidence to the existence of the PIERS effect depending on the charge transfer properties of the substrate.

Table S2: Calculation of the Enhancement Factor for different Ag nanoparticles' size distribution.

		Relative Intensity		EF		
Spectra	C (M)	1648(cm-1)	1358(cm-1)	1648(cm-1)	1358(cm-1)	Average EF
10s PIERS	10^{-5}	999	1027	6.71×10^4	1.41×10^5	1.04×10^5
10s SERS	10^{-5}	295	390	1.98×10^4	5.35×10^4	3.67×10^4
20s PIERS	10^{-5}	3815	4930	1.6×106	2.9×10^6	2.3×10^6
20s SERS	10^{-5}	730	642	3.1×10^5	3.7×10^5	3.4×10^5
40s PIERS	10^{-5}	4540	2063	3.05×10^5	2.83×10^5	2.94×10^5
40s SERS	10^{-5}	537	308	3.60×10^4	4.23×10^4	3.92×10^4
Bulk powder	2.75	4097	2004	1	1	1

Section III: Chemical characterization of PIERS and SERS substrates (XPS, TEM, Raman, XRD)

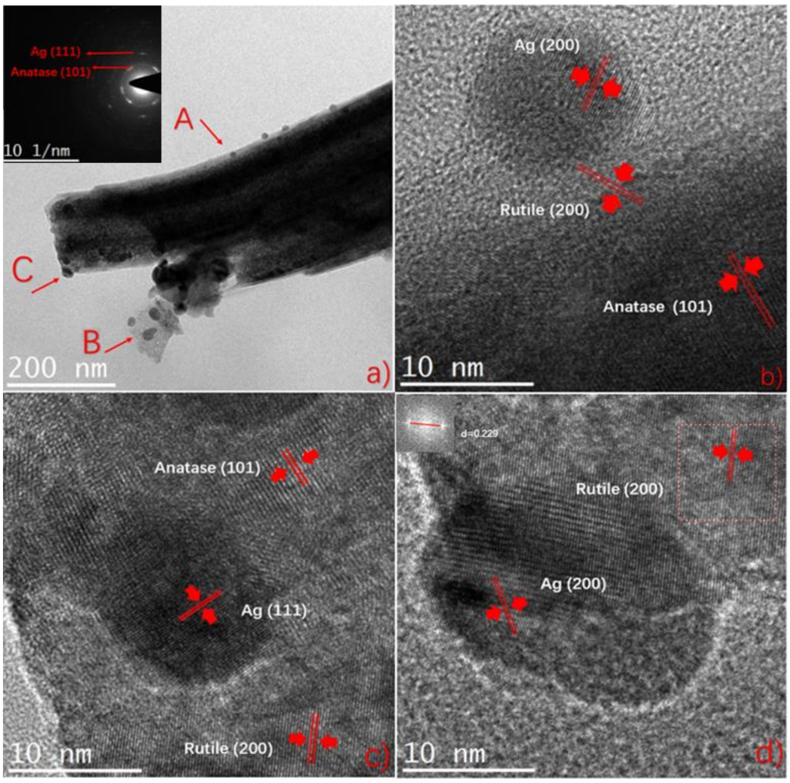

Figure S7 TEM characterization of PIERS substrate. Inset is the SAED diffraction assignment. A,B,C labels indicate different nanoparticles, and position A is shown in more detail in panel on top right (b).

The crystallographic *d*-spacing measured for Ag was 0.24 nm and 0.20 nm, which corresponds to the Ag (111) and Ag (200) planes. Most of the Ag nanoparticles were in contact with anatase (101) plane with a *d*-spacing of 0.35 nm and the rutile (200) plane with a *d*-spacing of 0.23 nm, in good agreement with previously reported values [3]. X-ray diffraction (XRD) in **Figure S8** confirms that the substrate consists of both rutile and anatase phases, in phase fractions 35% rutile to 65% anatase, determined via phase refinement techniques from the XRD data [4].

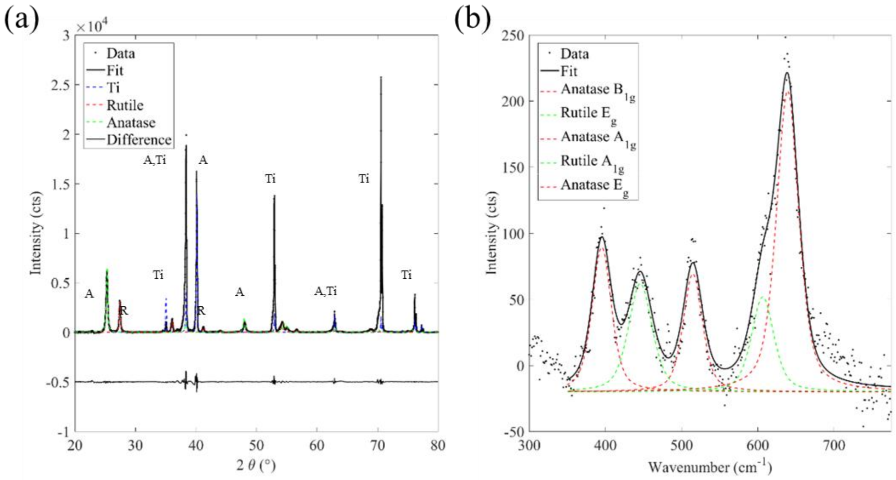

Figure S8 (a) X-ray diffractogram of PIERS substrate, with the residual non-Rietveld refined trace below, and bottom: Raman spectrum of PIERS substrate. (b) Raman spectra of PIERS substrate with peaks from

anatase and rutile phase fitted

To identify the surface crystal structure in contact with Ag nanoparticles and hence most relevant to the optical properties, we measured the Raman spectra of the bare PIERS substrate (**Figure S8**). The spectrum shows anatase TiO_2 peaks at 395, 515, and 639 cm^{-1} assigned to vibrations with B_{1g}, A_{1g} and E_g symmetry respectively [5]. However, there is still a clear rutile contribution at the shoulders of the main peaks at 446 and 606 cm^{-1}, corresponding to the E_g, and A_{1g} vibrations respectively [6]. Fitting the main peaks using the procedure in Ref [4] yields the phase fractions 29% rutile to 71% anatase, matching the bulk XRD refinement well. Unlike the PIERS substrate, the SERS substrate does not show any appreciable bulk crystallinity or the peaks associated with anatase or rutile - as expected due to the amorphous nature of as-anodized TiO_2 [7].

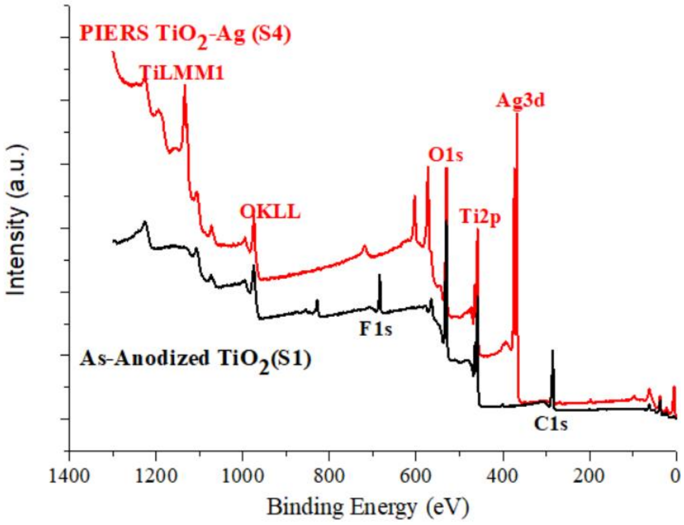

Figure S9 XPS survey Spectra for PIERS and As-anodized TiO2

We can also estimate the surface density of Ti(III) defects from the XPS data in Fig. 3b from the main text, which shows a ratio of Ti(III):Ti(IV) of 0.03. The density of anatase is 3.78 g/cm^3 with a molecular weight of 79.88 g/mol, giving a number density of 2.8×10^{22} cm^{-3}. Calculating a mean free path for Ti3p electrons of 2 nm yields a surface density Ti(III) defects of 1.7×10^{12} cm^{-2} = 17×10^3 μm^{-2}.

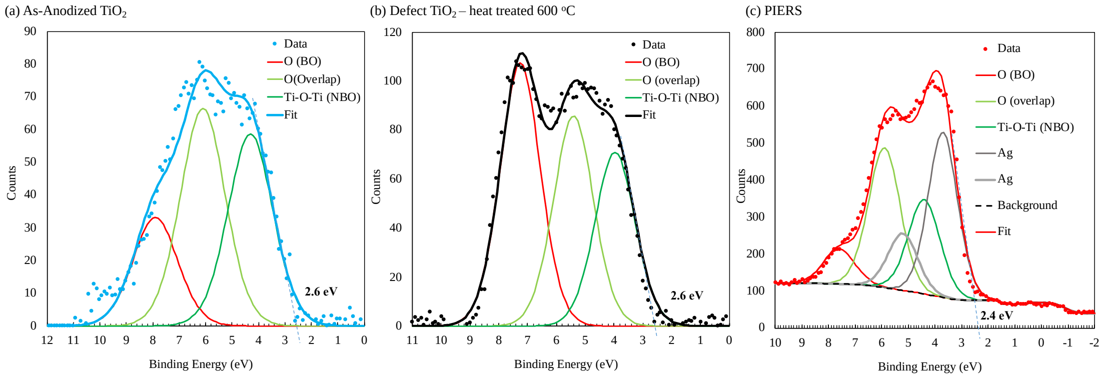

Figure S10 XPS Valence Band spectra and the extrapolated band edges for (a) As-anodized amorphous TiO2, (b) Defective crystalline TiO2, and (c) PIERS substrate

Section IV: Optical characterization of PIERS and SERS substrates

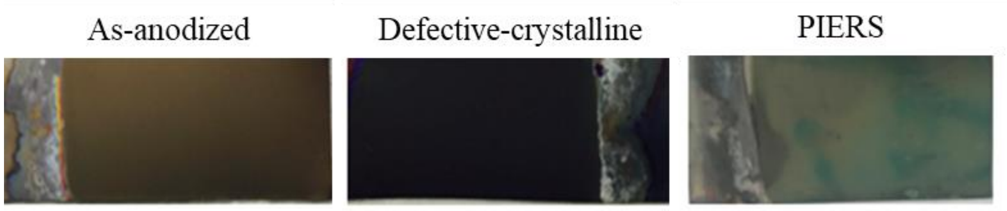

Figure S11 Optical image of substrates: As-anodized amorphous TiO_2, Defective crystalline TiO_2 (annealed at *600°C in Ar for 1 hour*), PIERS substrate with AgNPs

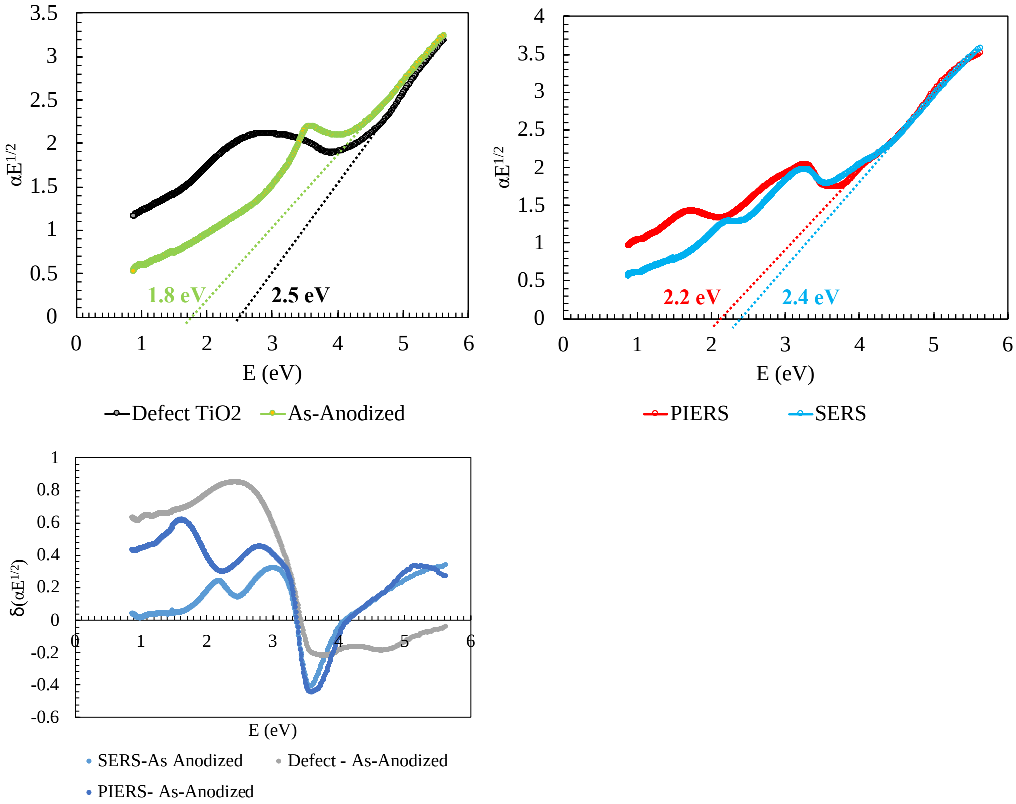

Figure S12 Kubelka-Munk function and Tauc Plots of the various samples used, along with subtraction spectra showing similarity between the SERS and PIERS samples in the region corresponding to the Ag plasmon resonances.

Section V: Raman peak fitting and analysis

Lack of contributions from amorphous carbon in subtraction spectra – spectral intensity changes hence due to PIERS and SERS effect.

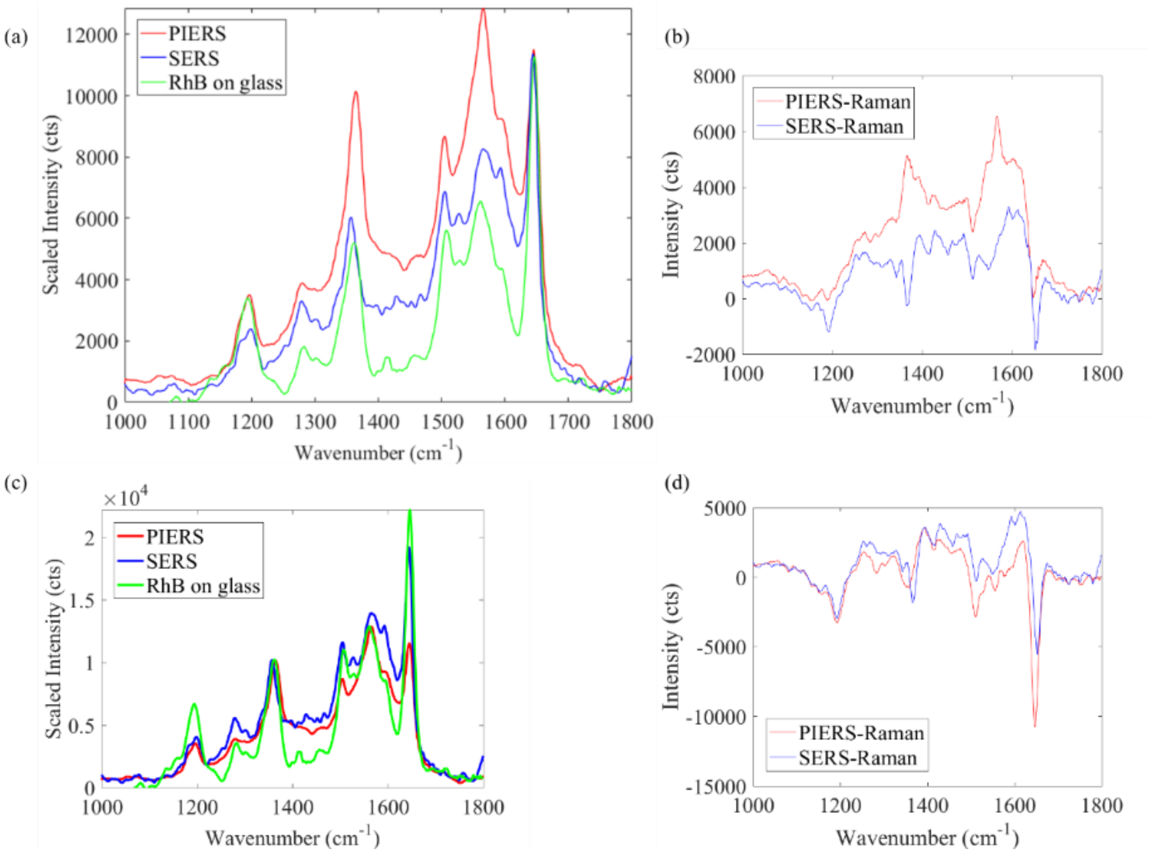

Figure S13 Raman difference spectra analysis. (a) Raman spectra scaled to the peak at ~1650 cm-1. (b) Subtraction spectra showing the source of the major differences of the spectra normalized to the peak at ~1650 cm-1. (c) Subtraction Raman spectra normalized to the peak at ~1650cm^{-1}. (d) Subtraction Raman spectra normalized to the peak at 1350cm^{-1}.

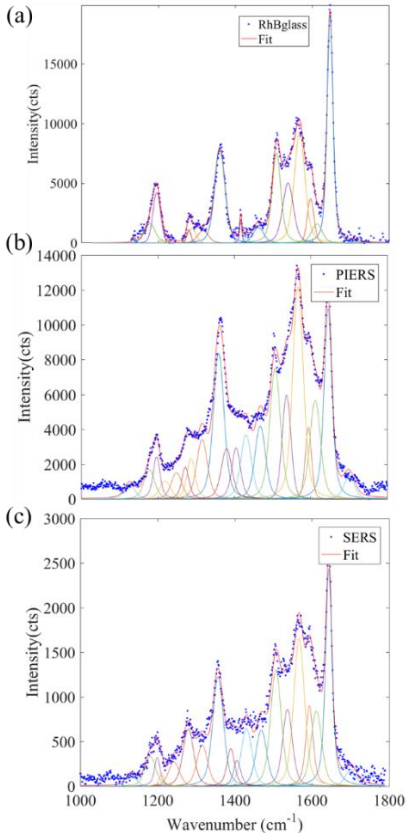

Figure S14 Curve resolution using a Voigt fucntion on linear background corrected Raman spectra collected at 10-5 M of RhB (a) RhB on aluminium foil on glass slides. (b) PIERS substrate (c) SERS substrate

Table S4: Raman peak fitting locations and peak assignments for different samples

Peaks normalized to intensity of main xanthene ring stretch peak at 1650 cm^{-1}.

Table S3: Raman spectral assignment for RhB

RhB cation	RhB powder			RhB on glass			SERS			PIERS		
**Assignment**	Peak (cm-1)	Intensity	Normalized Int	Peak (cm-1)	Intensity	Normalized Int	Peak (cm-1)	Intensity	Normalized In	Peak (cm-1)	Intensity	Normalized Int
**CM-H bend**							1075	0				
**XR puckering**	1081	0.22	0.28									
**CX-H in plane bend**	1131	0.11	0.14	1136	861.5	0.03	1132	170.7	0.03	1126	1234	0.07
**CX-CX stretch**	1183			1178	2364	0.08	1181	491.2	0.10	1178	2461	0.13
**CX-H bend**	1200	0.35	0.45	1196	5004	0.16	1199	801.5	0.16	1197	4658	0.25
				1210	765.8	0.02	1211	726	0.14	1219	1532	0.08
				1224	799.1	0.03	1243	401.8	0.08	1249	2221	0.12
				1279	1576	0.05	1280	939.6	0.19	1272	3420	0.18
**methyl wagging**	1282	0.66	0.85	1287	1451	0.05	1286	2.23E-14	0.00	1287	3946	0.21
**CX-H bend**	1311			1316	1540	0.05	1316	678.1	0.13	1316	5027	0.27
**CX-CX (xanthene ring) stretch**	1362	0.85	1.09	1360	1.09E+04	0.35	1357	1803	0.36	1359	1.23E+04	0.65
	1385	0.29	0.37	1380	2.68E-09	0.00	1390	744.4	0.15	1380	4244	0.22
**CX-N stretch**	1390											
				1414	4253	0.14	1406	545.6	0.11	1405	4789	0.25
**CX-CX stretch and CCX-H bend**	1426			1428	2026	0.06	1431	946.5	0.19	1431	5431	0.29
				1460	1940	0.06	1469	924.6	0.18	1468	6157	0.33
**CX-CX (xanthene ring) stretch**	1512	1	1.28	1507	1.24E+04	0.40	1507	1917	0.38	1507	1.10E+04	0.58
**CX-CX (xanthene ring) stretch**	1532	0.74	0.95	1537	7421	0.24	1537	1330	0.26	1537	6892	0.36
**CX-CX stretch**	1572	0.07	0.09	1567	1.35E+04	0.43	1567	2434	0.48	1567	1.79E+04	0.95
**CX-CX stretch**	1599	0.19	0.24	1596	4574	0.15	1594	1672	0.33	1594	5053	0.27
**CX-CX stretch**												
				1612	2378	0.08	1612	1238	0.25	1612	8331	0.44
**CX-CX (xanthene ring) stretch**	1649	0.78	1.00	1646	3.13E+04	1.00	1644	5032	1.00	1646	1.89E+04	1.00
				1711	157.4	0.01	1701	274.3	0.05	1701	1914	0.10
**RhB neutral**												
**carboxyl group that interacts with Cl**	1214											
**XR stretching**	1410											
**XR stretching**	1607											
**carboxyl group that interacts with Cl**	1706											

Section VI: Band alignment of semiconductor-metal-molecule

The alignment of the various energy levels was performed with the aid of the XPS and UV-Vis measurements. From measurements of the UV-Vis band edge of the defect TiO_2 sample, we can use the Kubelka-Monk function to obtain an optical gap of 2.5 eV (see Fig S12). We assign this optical gap to the energy difference from the occupied oxygen vacancy mid-gap state to the conduction band of TiO_2. We can estimate the overall bandgap of the TiO_2 mixed phase sample to be mainly that of the anatase phase at 3.2 eV [8]. From XPS measurements of the valence band spectrum (edge at binding energy of 2.6 eV), the valence band lies at 2.6 eV from the Fermi edge of silver (see Fig S10) due to Fermi level pinning between the Ag and semiconductor interface. As an additional check, the well-known redox couple of TiO_2 [9] has the valence band lie around -7.1 eV (2.7 V v.s. Standard Hydrogen Electrode) and the conduction band lie around -3.9 eV (-0.5 V v.s. Standard Hydrogen Electrode) below the vacuum level, consistent with our measured values with their uncertainties of ~0.1-0.3 eV, with differences reconciled due to band bending at the interface with silver. From the XPS calibration standard, we knew the vacuum level relative to the Fermi edge of silver, which placed silver's edge at 4.26 eV [10] above the Fermi edge . The adsorption of Rhodamine dyes can decrease the work function of Ag by 0.18 eV [11] - but this is very close to the XPS measurement uncertainty of 0.1 eV.

The alignment of RhB to the PIERS substrate can be performed using the well-known redox peaks of RhB in cyclic voltammetry (CV). Using the well-established onset potential method[12-15], we can relate the oxidation potential (E^0_{ox}) of RhB to its HOMO. RhB's E^0_{ox} can be identified from the CV in Fig. S15 below. A clear oxidation peak corresponding to RhB can be identified in the PIERS and bare Au electrodes, which is absent from the bare PIERS substrate in PBS solution. The value of E^0_{ox} = 0.89 V v.s. Ag/AgCl. Converting this to the standard hydrogen electrode and the vacuum potential can be done via[12, 16]: E_{HOMO} = -(E^0_{ox} + 4.28) = - 5.2 eV from the vacuum, placing it at 0.91 eV below the Fermi edge of Ag. This corresponds well to estimates from the literature placing the E_{HOMO} at -5.35 eV from the vacuum level, using the E°(RhB/RhB^+) redox couple at +0.95 V on the natural hydrogen electrode, and the E°(RhB*/RhB^+) redox couple at -1.42 V, relative to TiO_2 whose conduction band is at -0.5V and valence band is at +2.7V [17]

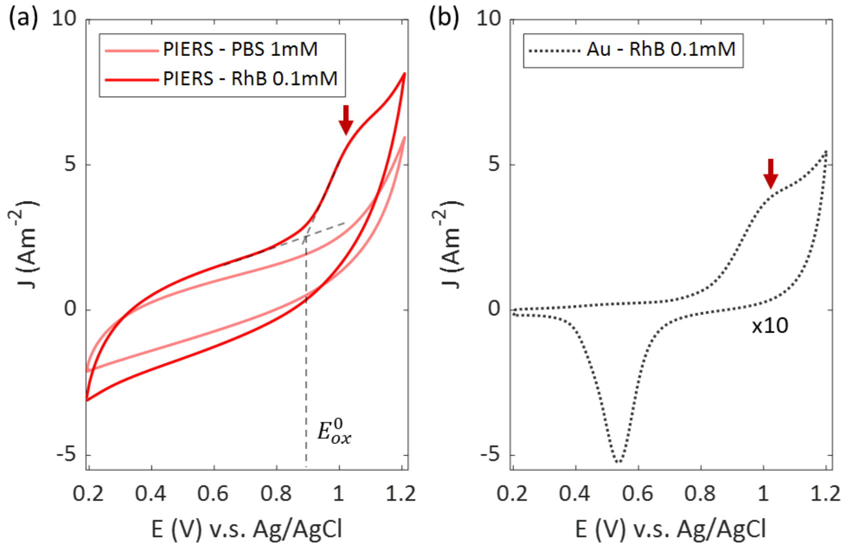

Figure S15 *Cyclic voltammogram of (a) PIERS substrate in phosphate-buffered saline (PBS, 1mM) and in*

aqueous Rhodamine B (RhB, 0.1 mM). (b) Evaporated Au on silicon electrode in RhB, with current scaled by 10x. All solutions were sparged in nitrogen gas flow before voltammograms were acquired at a scan rate of 25 mV/s. RhB redox peak indicated by an arrow.

Section VII: Time-Resolved Photoluminescence

Steady-state (Figure S16) and time-resolved PL spectra of the PIERS and SERS substrates with and without Ag NPs (Figure S17) were recorded by a gated intensified CCD camera (Andor Star DH740 CCI-010) connected to a grating spectrometer (Andor SR303i). The pulsed output from a mode-locked Ti:Sapphire optical amplifier (Spectra-Physics Solstice, 1.55 eV photon energy, 80 fs pulse width, 1 kHz repetition rate) was used to produce 400 nm excitation via second harmonic generation in a β-barium borate crystal. The iCCD gate (width 5 ns) was electronically stepped in 5 ns increments, relative to the pump pulse, to enable ns-temporal resolution of the PL decay.

Time-correlated single photon counting decay curves were obtained by exciting the PIERS and SERS substrates with and without Ag NPs with a Pico Quant LDH407 laser diode at 407 nm with a repetition rate of 40 MHz. The emission signal was selected with a monochromator to obtain the desired emission wavelength and detected by a Hamamastu R3809U-50 photomultiplier detector. Color filters (longpass filter) were utilized to re-move the scattered photons from the excitation laser. The decay curve is recorded for the emission at 460 nm. (Figure S18)

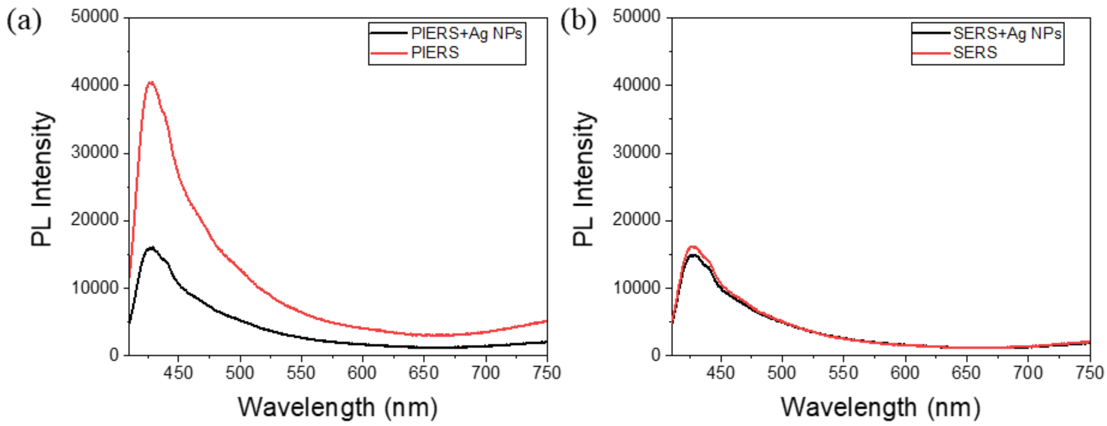

Figure S16 Steady State Photoluminescence Spectra. (a) PL of the PIERS substrates (Heat treated TiO_2) with and without Ag NPs. (b) PL of SERS substrates (As-anodized TiO_2 (No heat treatment)) with and without Ag NPs.

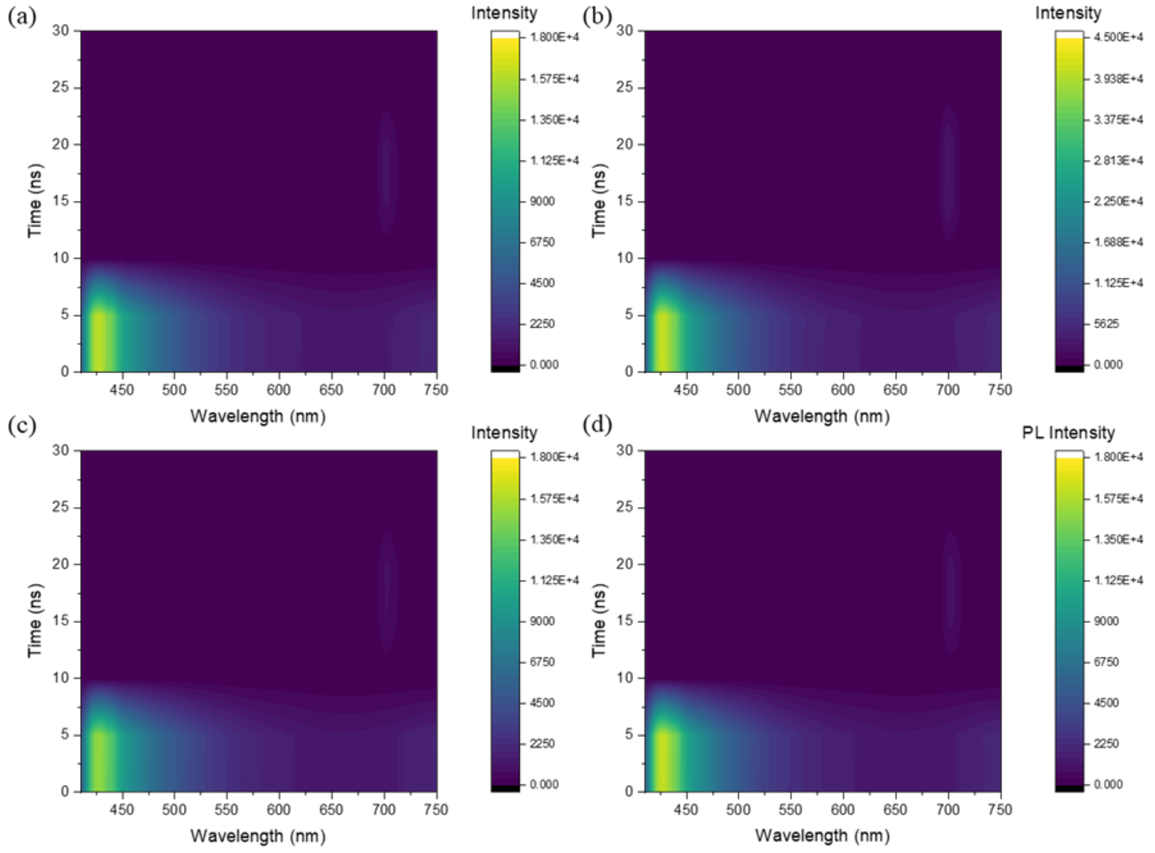

Figure S17. Transient Photoluminescence 2D Mapping. (a) PL of the PIERS substrates (Heat treated TiO_2) with Ag NPs. (b) PL of the PIERS substrates (Heat treated TiO_2) without Ag NPs. (c) PL of the SERS substrates (As-anodized TiO_2 (No heat treatment)) with Ag NPs. (d) PL of SERS substrates (As-anodized TiO_2 (No heat treatment)) without Ag NPs.

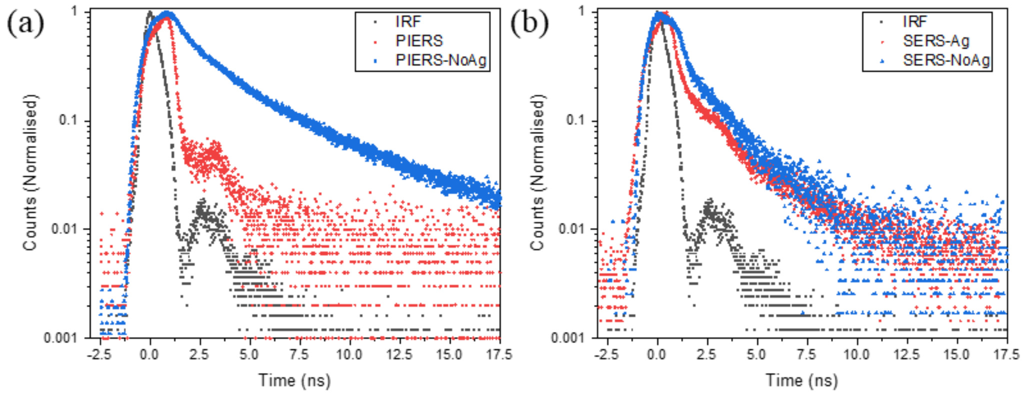

Figure S18. Time-correlated single photon counting at 460 nm emission. (a) PL decay of the PIERS substrates (Heat treated TiO_2) with and without Ag NPs and its instrument response function (IRF). (b) PL of SERS substrates (As-anodized TiO_2 (No heat treatment)) with and without Ag NPs and its internal response function (IRF).

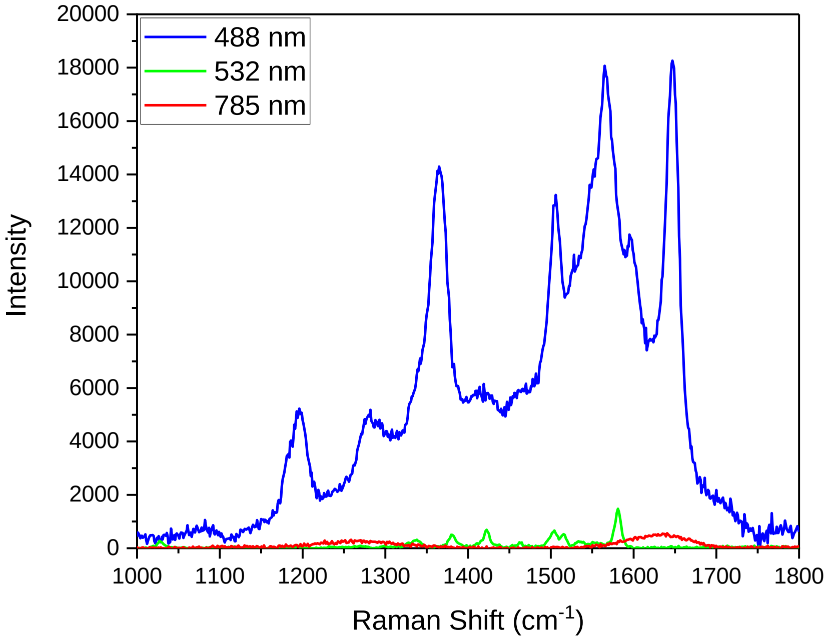

Figure S19 Excitation energy dependent Raman Spectra of RhB molecules. The Raman spectra are obtained under different pumping laser, including 488 nm, 532 nm and 785 nm.

Section VIII: Effect of annealing temperature on PIERS effect

By changing the annealing temperature with constant annealing treatment time (1 hr), we vary the amount of anatase v.s. rutile within the sample and the overall crystallinity[18]. This impacts the charge transfer process in PIERS upon photoexcitation, with greater crystallinity often improving charge transfer, showing an optimum at 600°C with a mixture of rutile to anatase. Past that, the PIERS enhancement decreases, due to changes in the charge transfer mechanism, which could be due to increasing concentration of the rutile phase which has a reduced charge diffusion length relative to anatase[19].

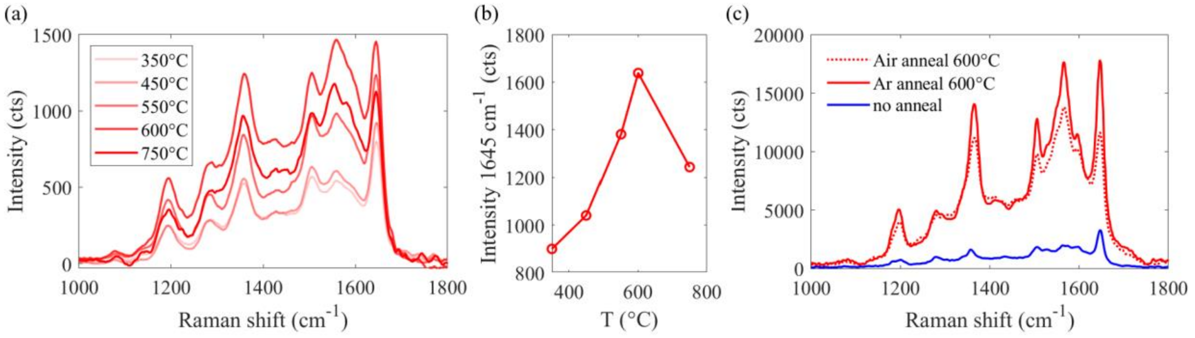

Fig. S20. PIERS enhancement relative to annealing temperature of TiO_2 (a) Raman spectra of PIERS substrates annealed at different temperatures (350-750°C) in Ar atmosphere. (b) Intensity of 1645 cm^{-1} peak v.s. annealing temperature. (c) Raman intensity difference between annealing in air v.s. Ar atmosphere at optimum 600°C.

Section IX: Effect of non-charge transfer compatible molecules on PIERS effect

PIERS and SERS spectra of 1-octanethiol (1mM solution in ethanol deposited on surface and dried) display little to no PIERS enhancement (main peak at 1595 cm^{-1} is only 20% higher, attributed to sample variation) due to misaligned energy levels for charge transfer with a deep HOMO level of -3.5 eV[20] from a metal's Fermi level and a HOMO-LUMO gap of 8-9 eV[21], which further supports this mechanism.

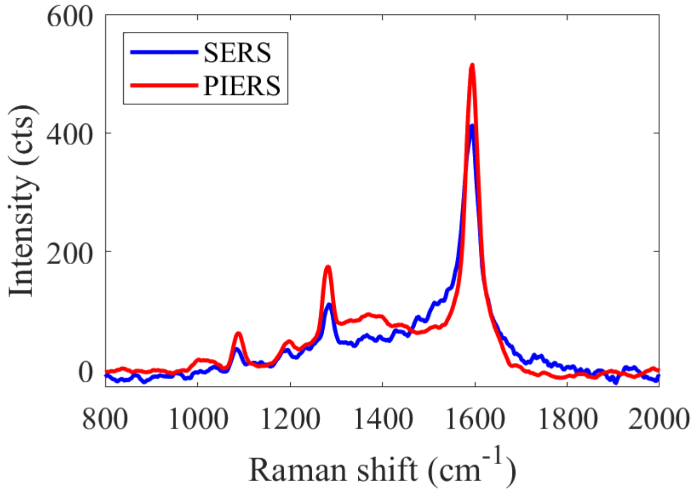

Fig. S21. Raman spectra of 1-octanethiol on SERS and PIERS substrates (collected with 20X objective, 488 nm, 0.03 mW power, 30s acquisition time, averaged over three sampling locations).

References

1. Le Ru, E.; Blackie, E.; Meyer, M.; Etchegoin, P. G., Surface enhanced Raman scattering enhancement factors: a comprehensive study. *The Journal of Physical Chemistry C* **2007,** *111* (37), 13794-13803.

2. Le Ru, E.; Etchegoin, P., *Principles of Surface-Enhanced Raman Spectroscopy: and related plasmonic effects*. Elsevier: 2008.

3. Duan, Y.; Zhang, M.; Wang, L.; Wang, F.; Yang, L.; Li, X.; Wang, C., Plasmonic Ag-TiO2−x nanocomposites for the photocatalytic removal of NO under visible light with high selectivity: The role of oxygen vacancies. *Applied Catalysis B: Environmental* **2017,** *204*, 67-77.

4. Zanatta, A., A fast-reliable methodology to estimate the concentration of rutile or anatase phases of TiO2. *AIP Advances* **2017,** *7* (7), 075201.

5. Frank, O.; Zukalova, M.; Laskova, B.; Kürti, J.; Koltai, J.; Kavan, L., Raman spectra of titanium dioxide (anatase, rutile) with identified oxygen isotopes (16, 17, 18). *Physical Chemistry Chemical Physics* **2012,** *14* (42), 14567-14572.

6. Challagulla, S.; Tarafder, K.; Ganesan, R.; Roy, S., Structure sensitive photocatalytic reduction of nitroarenes over TiO 2. *Scientific Reports* **2017,** *7* (1), 8783.

7. Dong, J.; Han, J.; Liu, Y.; Nakajima, A.; Matsushita, S.; Wei, S.; Gao, W., Defective black TiO2 synthesized via anodization for visible-light photocatalysis. *ACS Applied Materials & Interfaces* **2014,** *6* (3), 1385-1388.

8. Scanlon, D. O.; Dunnill, C. W.; Buckeridge, J.; Shevlin, S. A.; Logsdail, A. J.; Woodley, S. M.; Catlow, C. R. A.; Powell, M. J.; Palgrave, R. G.; Parkin, I. P., Band alignment of rutile and anatase TiO 2. *Nature Materials* **2013,** *12* (9), 798.

9. Hagfeldt, A.; Graetzel, M., Light-induced redox reactions in nanocrystalline systems. *Chemical Reviews* **1995,** *95* (1), 49-68.

10. He, J.; Iagher, L.; Etgar, L.; Avnir, D., Fine-tuning of the metal work function by molecular doping. *Chemical Communications* **2018,** *54* (52), 7203-7206.

11. Ciro, J.; Mesa, S.; Uribe, J. I.; Mejía-Escobar, M. A.; Ramirez, D.; Montoya, J. F.; Betancur, R.; Yoo, H.-S.; Park, N.-G.; Jaramillo, F., Optimization of the Ag/PCBM interface by a rhodamine

interlayer to enhance the efficiency and stability of perovskite solar cells. *Nanoscale* **2017,** *9* (27), 9440-9446.

12. Pepe, G.; Cole, J. M.; Waddell, P. G.; Perry, J. I., Rationalizing the suitability of rhodamines as chromophores in dye-sensitized solar cells: a systematic molecular design study. *Molecular Systems Design & Engineering* **2016,** *1* (4), 416-435.

13. Adeniyi, A. A.; Ngake, T. L.; Conradie, J., Cyclic Voltammetric Study of 2-Hydroxybenzophenone (HBP) Derivatives and the Correspondent Change in the Orbital Energy Levels in Different Solvents. *Electroanalysis* **2020,** *32* (12), 2659-2668.

14. Zhang, L.; Cole, J. M.; Dai, C., Variation in optoelectronic properties of azo dye-sensitized TiO2 semiconductor interfaces with different adsorption anchors: carboxylate, sulfonate, hydroxyl and pyridyl groups. *ACS Applied Materials & Interfaces* **2014,** *6* (10), 7535-7546.

15. Cole, J. M.; Pepe, G.; Al Bahri, O. K.; Cooper, C. B., Cosensitization in dye-sensitized solar cells. *Chemical Reviews* **2019,** *119* (12), 7279-7327.

16. Diggle, J.; Parker, A., Liquid junction potentials in electrochemical cells involving a dissimilar solvent junction. *Australian Journal of Chemistry* **1974,** *27* (8), 1617-1621.

17. Pan, L.; Zou, J.-J.; Liu, X.-Y.; Liu, X.-J.; Wang, S.; Zhang, X.; Wang, L., Visible–light–induced photodegradation of rhodamine B over hierarchical TiO2: effects of storage period and water-mediated adsorption switch. *Industrial & Engineering Chemistry Research* **2012,** *51* (39), 12782-12786.

18. Dong, J.; Ullal, R.; Han, J.; Wei, S.; Ouyang, X.; Dong, J.; Gao, W., Partially crystallized TiO 2 for microwave absorption. *Journal of Materials Chemistry A* **2015,** *3* (10), 5285-5288.

19. Luttrell, T.; Halpegamage, S.; Tao, J.; Kramer, A.; Sutter, E.; Batzill, M., Why is anatase a better photocatalyst than rutile?-Model studies on epitaxial TiO2 films. *Scientific Reports* **2014,** *4* (1), 4043.

20. Muntwiler, M.; Lindstrom, C.; Zhu, X.-Y., Delocalized electron resonance at the alkanethiolate self-assembled monolayer / Au (1 1 1) interface. *The Journal of Chemical Physics* **2006,** *124* (8), 081104.

21. Sotthewes, K.; Heimbuch, R.; Zandvliet, H. J., Manipulating transport through a single-molecule junction. *The Journal of Chemical Physics* **2013,** *139* (21), 214709.
