# Supplementary material for: Understanding the Chemical Mechanism behind Photoinduced Enhanced Raman Spectroscopy
Source: J Phys Chem Lett. 2023 May 11;14(19):4607–16. doi: 10.1021/acs.jpclett.3c00478 (PMC10201573; doi:10.1021/acs.jpclett.3c00478)
Supplement: Supplementary file 2 — jz3c00478_si_002.pdf [file jz3c00478_si_002.pdf]

jz-2023-00478r.R1

Name: Peer Review Information for "Understanding the Chemical Mechanism Behind Photo-Induced Enhanced Raman Spectroscopy"

First Round of Reviewer Comments

Reviewer: 1

Comments to the Author

1. What is the major advance reported in the paper?

The major technical advance claimed here is elucidation of the mechanism underlying phot-induced enhanced Raman spectroscopy (PIERS). Specifically, the authors claim that excitation of oxygen vacancy sites leads to a cascade of processes ending with charge transfer to the adsorbed dye (RhB) molecule.

The authors employ an impressive array of different experimental techniques to elucidate the mechanism but given the complexity of the system under study most of the evidence presented falls into the “circumstantial” category. There is no doubt that a significant Raman enhancement is observed by creating nanoparticle substrates on defect containing crystalline TiO2. However, the final conclusion of a charge transfer process is complicated by several factors: lack of independent verification of the RhB surface adsorption geometry and the diffuse appearance of the RhB spectra.

2. What is the immediate significance of this advance?

An interesting and thought-provoking claim in the conclusion is: now that we understand the mechanism we can rationally design systems to use the PIERS mechanism. This claim would be more convincing if the authors demonstrated this with another molecule.

As an aside, no rationale is given for choosing RhB. For example, why not try R6G - the spectral signatures are better separated and more work has been done on the adsorption geometry, although significant disagreements still exist in the literature on this point. This is one of the reasons for my suspicion surrounding the surface geometry proposed here. It is also likely that a mix of states exists. Alternatively, can the authors show that other standard SERS probes (e.g. benzene thiol) do or do not exhibit PIERS and rationalize this with their proposed mechanisms?

3. Technical suggestions

1) This article reads at times more like a perspective or a full-length article, not a letter. Extensive use of the SI is made in the main text and I found myself constantly going back and forth between the two. I think the authors should reconsider what needs to be in SI and what needs to be in the main narrative.

2) I'm not sure if it's a typo or not, but I don't understand the reference to 1518 on p8 l46 left column. If I look at Figure 5 the band that appears to increase in the charge transfer amine complex is around 1600, but the vibrational mode plotted at the top is 1518.

3) Given the variability in SERS enhancements, sometimes even within a spectra, it is difficult to believe that the relative line intensities in Red and Blue in Figure 5a should be interpreted as clear evidence of charge transfer. (For example, the wavelength dependent enhancement factors of the substrate could simply change by a little bit in this range and it would lead to the - not large - differences in band intensities relative to the massive (10^6) PIERS/SERS enhancements.

4) Given that the authors rely on electrochemical measurements for some of their study, it seems that measuring changes in the spectra under an applied potential could significantly strengthen their claims.

5) pH plays a large role in binding geometry and SERS spectra for compounds such as RhB. The authors should comment on this in their manuscript and state what, if anything, they did to control for it. For example, experiments could be done in the presence of buffered solutions of different pH to further test their hypotheses.

5) Figure S6 – Caption is missing descriptions for panels (b) and (c)

6) Figure S13 – Caption is missing description for panel (d)

7) Figure S17 – I believe the x-axis label is incorrect on (d)

Overall, the amount of work here is impressive, but the system is very complicated making a clear attribution of the mechanism difficult.

Reviewer: 2

Comments to the Author

This manuscript reinvestigates the origin of photo-induced enhanced Raman scattering (PIERS), suggesting that, contrary to the hypotheses proposed so far, at the basis of this effect there is a significant increase of Raman polarizability caused by photonic excitation.

These results offer a new interpretation of PIERS. Previous literature associated the effect to the increase of electron density nearby the noble metal nanoparticles, which is assisted by titanium vacancy sub-band gap energy levels introduced upon UV irradiation. Going more into details, the main steps of PIERS generation are actually the same as proposed in previous literature, i.e.: (i) absorption of visible light by Ti vacancy defect states; (ii) electron transfer from defect states to the conduction band of TiO_2, (iii) electron transfer to the Fermi level of plasmonic nanoparticle and (iv) electron transfer from plasmonic nanoparticle to the adsorbed analyte. According to DFT calculations reported in this work, it would be the latter step that determines the increase of molecular polarizability and, eventually, the enhanced Raman response.

Thus, this study suggests that PIERS is mainly due to a chemical, not to a merely electromagnetic enhancement, and this chemical enhancement is not caused by metal-to-ligand type charge transfer, but

it is due to the bond between metal nanoparticles (fed by electrons from TiO2 upon irradiation) and the analyte.

This conclusion is interesting, however, to make it more convincing, I would suggest addressing the following points:

1) The authors compared defective TiO2, i.e. annealed in argon at high temperature, with as prepared, amorphous titania. It would be more informative to compare the PIERS substrate (based on defective TiO2) with another TiO2 substrate annealed in air at the same temperature (400°). In this way the effect of titania oxygen vacancies would be clearly elicited.

2) Related to the previous point, at Page 4, the statement "a controlled amount of oxygen defects…" suggests that there is a full control of oxygen defects and their density. However, in the remaining part of the manuscript there are neither direct evidence of this control nor any quantification of defect density. XPS data only give the status of the sample, but the key point is to develop a protocol to precisely control the amount of Ti3+ in PIERS substrates. This is mandatory, in view of developing a tool that is relevant for sensing and chemical analysis. I would suggest to better clarify this point, as the oxygen defect states are the cornerstone of the whole PIERS effect.

3) Raman experiments should be run very carefully, in order to achieve reliable data. In particular, data should be acquired avoiding probing regions with local accumulations of the analyte and a statistically relevant number of regions should be analyzed, with an indication of relative standard deviation. A precise description of data acquisition and analysis is missing and should be added.

4) Supporting Information. Figure S2 shows that the Raman detection of RhB is strongly dependent on sputtering time, which means that there is a dependence on the density of distribution of silver clusters. Is this experimental observation coherent with the mechanism proposed? In other words, is polarizability affected by the nanoparticle size? This aspect is not clear, as nanoparticle size seems to provide a greater contribution to Raman enhancement.

5) Page 8: "The PIERS substrate showed a 580% enhancement of Raman signals than the equivalent SERS substrate without defective crystalline TiO2". How do you calculate it?

6) Introduction (page 2): ..."these techniques have not achieved comparable SERS enhancement or work as reproducibly as metal nanoparticle-based SERS". This statement is questionable and somehow misleading. Literature of last ten years reports many examples showing that all-dielectric SERS active substrates are more reproducible and sensitive than plasmonic SERS (Chem. Rev 2016, https://doi.org/10.1021/acs.chemrev.6b00365; J. Raman Spectrosc. https://doi.org/10.1002/jrs.4854; J. Raman Spectrosc. https://doi.org/10.1002/jrs.5330; Nat. Commun. 6, 7800 (2015)).

Moreover, they are less invasive and allow for monitoring chemical reactions with minimal perturbation. (JACS 2013 https://doi.org/10.1021/ja401666p; Small 2014 https://doi.org/10.1002/smll.201303166, Nature Commun. 2018, https://doi.org/10.1038/s41467-018-05237-x)

Minor:

Please check some typos, for example page 7, row 29, right column: "florescence"

Author's Response to Peer Review Comments:

Response to reviewers

We thank the editor and reviewers for their insightful comments and the opportunity to improve the manuscript. We greatly appreciate the feedback from all reviewers who emphasise that the manuscript employs "an impressive array of different experimental techniques to elucidate the mechanism" and that we propose a mechanism of photo-induced enhanced Raman spectroscopy (PIERS) "contrary to the hypotheses proposed so far" with an "interesting and thought-provoking claim in the conclusion" and conclude that the "amount of work here is impressive".

The mechanism of PIERS is multi-faceted, which we highlight in our introduction and as pointed out by the reviewers. While previous works have proposed various hypotheses[1], they lack detailed time-resolved spectroscopic, structural, and electrochemical evidence supporting it. Using a wide range of tools, we have performed the *first* mechanistic determination of the energy levels and kinetics relevant in PIERS for a model molecule: Rhodamine B. We have contributed a key new piece of the puzzle in terms of dynamic polarizability changes due to charge transfer that must be considered. Additionally, we apply structural tools such as high-resolution TEM and XRD to determine the role of crystallinity and defect density on the charge transfer steps necessary for PIERS enhancement, for the first time. We believe this understanding is generalizable to other molecular systems, and now provide new experiments:

- Raman spectra for a molecule whose energy level alignment is unfavourable for PIERS, yet acts as a standard Raman probe – 1-octanethiol[2],
- PIERS spectra testing the effect of annealing temperatures,
- PIERS spectra in different annealing environments (air v.s. Ar) to clarify the role of oxygen vacancies.

Our work not only tests a hypothesis for the final step of the PIERS enhancement, but also excludes three other hypotheses (charge transfer resonance Raman, increased plasmonic enhancement, and fluorescence quenching). We believe this is a significant contribution to determining the mechanism of the PIERS process.

We have highlighted all changes in the main manuscript and supplementary information in red.

Reviewer #1:

"The major technical advance claimed here is elucidation of the mechanism underlying phot-induced enhanced Raman spectroscopy (PIERS). Specifically, the authors claim that excitation of oxygen vacancy sites leads to a cascade of processes ending with charge transfer to the adsorbed dye (RhB) molecule. The authors employ an impressive array of different experimental techniques to elucidate the mechanism but given the complexity of the system under study most of the evidence presented falls into the "circumstantial" category. There is no doubt that a significant Raman enhancement is observed by creating nanoparticle substrates on defect containing crystalline TiO2. However, the final conclusion of a charge transfer process is complicated by several factors: lack of independent verification of the RhB surface adsorption geometry and the diffuse appearance of the RhB spectra."

We thank the reviewer for highlighting the array of experimental techniques used, however we disagree that the evidence is circumstantial. We provide, for the first time, direct evidence of the kinetics of the previously conjectured photo-induced charge transfer of an excited electron from the defect states of TiO_2 to Ag using static spectroscopy and time-resolved nanosecond

photoluminescence spectroscopy. Furthermore, we showed using X-ray photoelectron spectroscopy (XPS) the shift in binding energies of Ag and Ti that indicates static interfacial charge transfer.

In the final step of charge transfer enhancing the Raman polarizability of RhB, our conclusion relies on a comparison of the energy levels measured independently through cyclic voltammetry to show that such a charge transfer is energetically favourable, and also through a comparison of the Raman spectra to DFT calculations. The DFT calculations of Ag adsorption energy at several sites and an application of the Moskovitz selection rules for enhanced Raman[3], as mentioned in the main text, allow us to assign the predominant orientations of RhB on the surface. Indeed, there is likely a mixture of orientations. *The Raman measurement technique is only sensitive to some of these orientations*, due to the different Raman polarizabilities of different binding geometries, and SERS/PIERS preferentially providing signals from the ones with the highest polarizabilities. In all cases calculated, the coordination of RhB to Ag increases the Raman polarizability, except for coordination of RhB's xanthene moiety to Ag; this observation further supports our conclusion that the interaction of Ag with RhB occurs via the nitrogen atom.

We modify the main text below to highlight this:

"The most likely binding geometry was determined by calculating the Raman modes of RhB+ using DFT in Fig. 5(a) (B3PW91/6-31G+(d,p) and LANL2DZ(for Ag)), and applying Moscovitz's SERS surface selection rules[64]: normal modes with a large polarizability component normal to the metal's surface will be enhanced. In Fig. 5(a), both the PIERS and SERS substrates show an enhanced Raman intensity of the antisymmetric xanthene ring stretch at ~1370 cm^{-1} (theory: 1344 cm^{-1}) and symmetric amine stretches at ~1200 cm^{-1} (theory: 1260cm^{-1}) relative to the pure xanthene ring stretch at 1646 cm^{-1}. As the enhanced xanthene ring stretches have their displacement vectors aligned along the long axis of the RhB molecule (Fig. 5(c)), this implies that the binding needs to occur via a functional group face that is aligned to RhB's long axis. This leaves only one option: the nitrogen fragment of the RhB must bind strongly to the Ag surface. This conclusion is supported by the increased intensity of peaks associated with a xanthene ring carbon-nitrogen stretch (1380-1390 cm^{-1}). If RhB were lying fully flat, it would be the out-of-plane modes that would be enhanced, at the expense of the in-plane modes – which is not observed. Marchi et. al.[59] also found that RhB prefers to coordinate to Ag via its amine groups. As the amine's motion is hindered, the fluorescence quantum yield should increase as the greater rigidity reduces the rate of internal conversion – which we observe as an overall increase in the Raman background (Figure S3). However, the absolute determination of the range of likely binding geometries is challenging, and dependent on nuances such as local pH. Despite that, all simulations with Ag present increase the Raman polarizability of RhB, except for coordination to the xanthene, which further supports the coordination of Ag to N dominating the SERS and PIERS spectra."

The surface selection rules allow us to assign the predominant orientation of RhB on the surface. However, we agree that there are further steps to determining the absolute orientation and indeed the range of orientations that RhB must have on the surface of Ag and have updated the text below to reflect this. Further ultrafast spectroscopic techniques such as polarized surface-enhanced sum-frequency generation spectroscopy[4] and surface-enhanced hyper-Raman spectroscopy[5] may determine the orientation, however such experiments are outside of the scope of this letter. We emphasize that we are not explicitly determining all the possible geometries of RhB binding on an Ag surface, and only highlight the few likely to be relevant to the PIERS enhancement process.

"An interesting and thought-provoking claim in the conclusion is: now that we understand the mechanism we can rationally design systems to use the PIERS mechanism. This claim would be more convincing if the authors demonstrated this with another molecule.

As an aside, no rationale is given for choosing RhB. For example, why not try R6G - the spectral signatures are better separated and more work has been done on the adsorption geometry, although significant disagreements still exist in the literature on this point. This is one of the reasons for my suspicion surrounding the surface geometry proposed here. It is also likely that a mix of states exists. Alternatively, can the authors show that other standard SERS probes (e.g. benzene thiol) do or do not exhibit PIERS and rationalize this with their proposed mechanisms?"

We thank the reviewer for recognizing our interesting conclusions. While more has been done to determine the binding of Rhodamine-6-G (R6G), at the laser wavelength used to excite the PIERS process (488 nm), R6G possess strong absorption bands (Fig. R1) unlike RhB. This will give rise to a large fluorescence background and resonance Raman enhancement which would complicate disentangling the true contribution of the PIERS mechanism. Hence, we do not perform experiments with R6G.

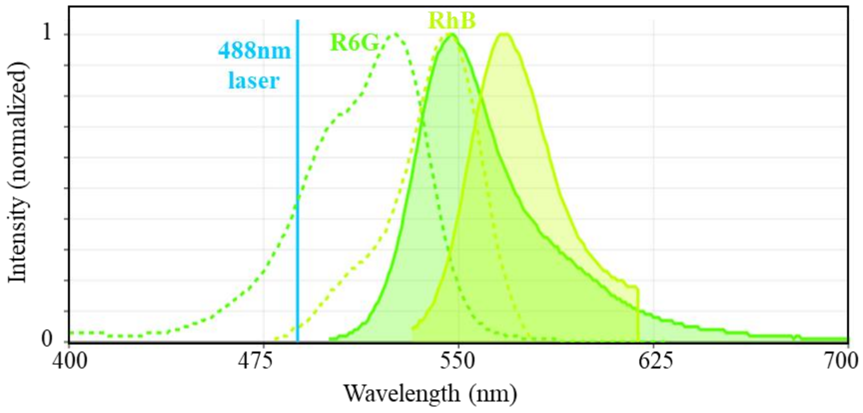

Fig. R1. Absorption (dotted lines) – emission (solid shaded line) spectrum of R6G (dark green) and RhB (light green) relative to the laser excitation wavelength at 488 nm. (spectra from Ref.[6])

This is a key contribution of our work, as previous PIERS works[7-15] use dye molecules with strong resonance Raman enhancement exactly at the laser excitation wavelength. We highlight this further in the introduction:

"Prior works have proposed a charge-transfer based enhancement for many dye molecules in pure/defect engineered semiconductors[14-21], semiconductor heterostructures[22, 23] or semiconductor-metal heterostructures[24]. However, such works have used conditions in which the dye molecule (such as Rhodamine-6-G) is on- or near-resonance with the excitation laser[14, 16, 19, 23, 25-29], thus having resonance Raman enhancements in addition to substrate-induced enhancements, or claim charge transfer without providing evidence for the exact mechanism [17, 18, 30]. Indeed only a few studies [14, 20, 24, 27, 31, 32] show evidence that bona-fide charge transfer resonances between localized molecular states to semiconductor bands are boosting the observed Raman signals, while others[33, 34] suggest this but do not provide any direct evidence of energetically favorable band alignments. No effort has been made yet to map out the energy levels involved to establish the mechanism for Raman signal enhancement by PIERS."

Future work will measure multiple molecules. However, for this work, the full range of analytical techniques used (cyclic voltammetry, nanosecond time resolved spectroscopy, x-ray photoelectron spectroscopy, etc.) have only been performed on one model molecule (RhB).

We have performed a **new experiment** using 1-octanethiol which we share below. This system displays little to no PIERS enhancement (main peak at 1595 cm^{-1} is only 20% higher, attributed to sample variation) due to misaligned energy levels for charge transfer with a deep HOMO level of -3.5 eV[16] from a metal's Fermi level and a HOMO-LUMO gap of 8-9 eV[17].This new data further supports our proposed mechanism for PIERS. We now include this as a discussion in the main text page 7, and in SI Section S9.

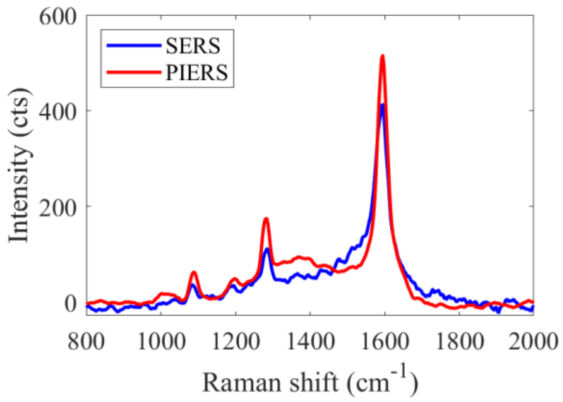

Fig. R2. Raman spectra of 1-octanethiol on SERS and PIERS substrates (collected with 20X objective, 488 nm, 0.03 mW power, 30s acquisition time, averaged over three sampling locations).

3. Technical suggestions

1) This article reads at times more like a perspective or a full-length article, not a letter. Extensive use of the SI is made in the main text and I found myself constantly going back and forth between the two. I think the authors should reconsider what needs to be in SI and what needs to be in the main narrative.

We thank the reviewers and make changes to streamline references to the SI in the main text. We emphasize that our extensive SI is to provides additional information/control studies for our mechanism. All critical information is contained in the main figures.

2) I'm not sure if it's a typo or not, but I don't understand the reference to 1518 on p8 l46 left column. If I look at Figure 5 the band that appears to increase in the charge transfer amine complex is around 1600, but the vibrational mode plotted at the top is 1518.

We thank the reviewer for pointing this out and have corrected this in the manuscript. Indeed, it was a typo; the mode of interest is at 1580 cm^{-1}.

3) Given the variability in SERS enhancements, sometimes even within a spectra, it is difficult to believe that the relative line intensities in Red and Blue in Figure 5a should be interpreted as clear evidence of charge transfer. (For example, the wavelength dependent enhancement factors of the substrate could

simply change by a little bit in this range and it would lead to the - not large - differences in band intensities relative to the massive (10^6) PIERS/SERS enhancements.

We show consistent replicate SERS measurements across the sample, showing similar relative intensity changes in Supplementary Section S1, with consistent enhancement of peaks at 1580 cm^{-1} in the PIERS relative to the SERS substrate. We also highlight our measurement protocol in response to Reviewer #2's third question below, and show data at different concentrations of RhB sampled in different locations.

While we agree that SERS measurements can be variable on certain substrates and single nanostructures, in these measurements, we took care to measure with a low numerical aperture objective lens, averaging spectra over an area several microns across in multiple measurements. Furthermore, the conclusion of static charge transfer leading to an increased Raman polarizability is supported through cyclic voltammetry measurements.

Our work not only offers a good hypothesis for the final step of the PIERS enhancement, but also examines and excludes three other hypotheses (charge transfer resonance Raman, increased plasmonic enhancement, and fluorescence quenching). Other explanations are possible, but we propose the one most consistent with available evidence.

4) Given that the authors rely on electrochemical measurements for some of their study, it seems that measuring changes in the spectra under an applied potential could significantly strengthen their claims.

We use the electrochemical measurement to determine the HOMO and LUMO levels of RhB on the PIERS substrate surface. For the PIERS mechanism however, all charge transfer is photo-induced by laser excitation, hence an applied external potential would not reproduce the same effect due to a lack of plasmonic *optical* field localization driving external charge transfer[18, 19] through e.g. hot electrons. However, this could be useful for tuning the molecule's HOMO and LUMO levels statically, which is an excellent suggestion. Efforts to build a custom microscope-compatible spectro-electrochemical cell are underway, however it is outside the scope of this work.

5) pH plays a large role in binding geometry and SERS spectra for compounds such as RhB. The authors should comment on this in their manuscript and state what, if anything, they did to control for it. For example, experiments could be done in the presence of buffered solutions of different pH to further test their hypotheses.

All samples are dry during measurement and RhB was deposited on the surface from an ethanol solvent and subsequently dried. Indeed, there are changes in the protonation state of RhB upon binding to the surface, which is hard to control on the PIERS substrate. Since samples are dry in most experiments of PIERS thus far[1, 20], control of pH using a buffer is difficult. We now discuss these nuances in the main text:

"The absolute determination of binding geometry is challenging, and dependent on nuances such as local pH."

6) Figure S6 – Caption is missing descriptions for panels (b) and (c)

7) Figure S13 – Caption is missing description for panel (d)

8) Figure S17 – I believe the x-axis label is incorrect on (d)

We are grateful to the reviewer for noticing these and have implemented these corrections.

Reviewer #2:

This manuscript reinvestigates the origin of photo-induced enhanced Raman scattering (PIERS), suggesting that, contrary to the hypotheses proposed so far, at the basis of this effect there is a significant increase of Raman polarizability caused by photonic excitation.

These results offer a new interpretation of PIERS. Previous literature associated the effect to the increase of electron density nearby the noble metal nanoparticles, which is assisted by titanium vacancy sub-band gap energy levels introduced upon UV irradiation. Going more into details, the main steps of PIERS generation are actually the same as proposed in previous literature, i.e.: (i) absorption of visible light by Ti vacancy defect states; (ii) electron transfer from defect states to the conduction band of TiO2, (iii) electron transfer to the Fermi level of plasmonic nanoparticle and (iv) electron transfer from plasmonic nanoparticle to the adsorbed analyte. According to DFT calculations reported in this work, it would be the latter step that determines the increase of molecular polarizability and, eventually, the enhanced Raman response.

Thus, this study suggests that PIERS is mainly due to a chemical, not to a merely electromagnetic enhancement, and this chemical enhancement is not caused by metal-to-ligand type charge transfer, but it is due to the bond between metal nanoparticles (fed by electrons from TiO2 upon irradiation) and the analyte.

We thank the reviewer for their positive comments and highlight that while the initial steps are consistent with that proposed in the wider literature, we now show actual spectroscopic and time-resolved nanosecond kinetic evidence of the PIERS process, which was not measured before.

1) The authors compared defective TiO2, i.e. annealed in argon at high temperature, with as prepared, amorphous titania. It would be more informative to compare the PIERS substrate (based on defective TiO2) with another TiO2 substrate annealed in air at the same temperature (400°). In this way the effect of titania oxygen vacancies would be clearly elicited.

In response to this reviewer's helpful observation, we report **new experimental results** showing the effect of the different annealing temperatures and environments within the SI (section S8). In the main text, we highlight the role of defects, which increase visible laser absorption at the cost of reduced charge transfer mobilities. A balance between crystallinity and defect concentration is needed. We now also find an optimum annealing temperature in Ar atmosphere of 600°C (Fig. R3b) for PIERS enhancement. At this optimum temperature, annealing in an oxygen-deficient Ar atmosphere v.s. air (Fig. R3c) is critical for higher PIERS enhancements. We now include an additional discussion in the SI Section S8:

"By changing the annealing temperature with constant annealing treatment time (1 hr), we vary the amount of anatase v.s. rutile within the sample and the overall crystallinity[21]. This impacts the charge transfer process in PIERS upon photoexcitation, with greater crystallinity often improving charge transfer, showing an optimum at 600°C with a mixture of rutile to anatase. Past that, the PIERS

enhancement decreases, due to changes in the charge transfer mechanism, which could be due to increasing concentration of the rutile phase which has a reduced charge diffusion length relative to anatase[22], and also a decreased absorption of light as defect density is reduced."

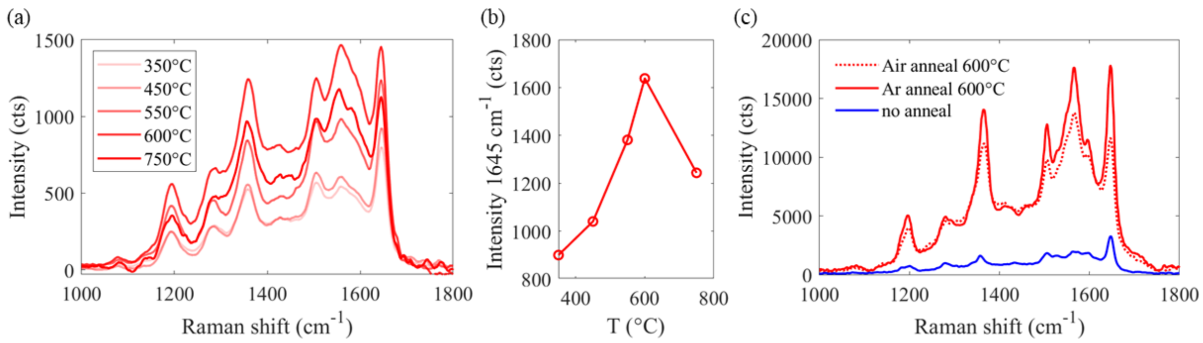

Fig. R3. PIERS enhancement relative to annealing temperature of TiO_2 (a) Raman spectra of PIERS substrates annealed at different temperatures (350-750°C) in Ar atmosphere. (b) Intensity of 1645 cm^{-1} peak v.s. annealing temperature. (c) Raman intensity difference between annealing in air v.s. Ar atmosphere at optimum 600°C.

2) Related to the previous point, at Page 4, the statement "a controlled amount of oxygen defects…" suggests that there is a full control of oxygen defects and their density. However, in the remaining part of the manuscript there are neither direct evidence of this control nor any quantification of defect density. XPS data only give the status of the sample, but the key point is to develop a protocol to precisely control the amount of Ti3+ in PIERS substrates. This is mandatory, in view of developing a tool that is relevant for sensing and chemical analysis. I would suggest to better clarify this point, as the oxygen defect states are the cornerstone of the whole PIERS effect.

We now estimate the defect density from XPS measurements as 17 x10^3 μm^{-2} (details now included in SI section S3) and included a discussion in the text citing our previous work[21, 23] on controlling defect density in TiO_2 and crystallinity, along with the new work showing that annealing temperature controls the PIERS enhancement magnitude, with an optimum in annealing temperature. We emphasize further the role of balance between the increased crystallinity required for efficient charge transfer, and increased defect density which improves light absorption yet reduces charge transfer efficiency. This was also investigated by Glass et. al.[20], and we emphasize that finding exact defect densities of Ti^{3+} is non-trivial, as it requires positron annihilation lifetime spectroscopy[24] which is not a readily available lab-based technique and is a bulk measurement often requiring a large quantity of material. In the main text page 4, we now add:

"Changing the crystallinity through different annealing temperatures also reveals an optimum in PIERS enhancement, due to increasing rutile fraction with lower charge mobility and decreased defect-assisted light absorption, further supporting this hypothesis (SI Section S8)."

3) Raman experiments should be run very carefully, in order to achieve reliable data. In particular, data should be acquired avoiding probing regions with local accumulations of the analyte and a statistically relevant number of regions should be analyzed, with an indication of relative standard deviation. A precise description of data acquisition and analysis is missing and should be added.

We thank the reviewer for highlighting the importance of sampling in Raman spectroscopy experiments, which we have taken into consideration by performing replicate experiments across our samples and have now included further details in the methods section as below:

"Raman spectra were acquired using a Renishaw RM1000 Raman Microprobe comprising a single grating spectrograph of 2400 g/mm with a holographic notch filter removing Rayleigh scattered light below 150 cm^{-1}, with a 20 × objective lens (N.A. 0.5) providing a spot size at the surface of 1 – 2 µm and entrance slit width of 50 µm. Excitation at 488 nm was provided by an air-cooled Spectra-Physics Argon ion laser at 1 mW. The test molecule used in this study was Rhodamine B (RhB). The acquisition time was 60 sec per spectrum with 3 accumulations. For tests on the PIERS and SERS substrates, ~5 µL of RhB solution (10^{-5} $molL^{-1}$, 10^{-6} $molL^{-1}$, and 10^{-7} $molL^{-1}$) was deposited onto each substrate and dried in the air for 5 min prior to Raman spectra acquisition. All spectra presented are collected over at least 3 locations and averaged."

4) Supporting Information. Figure S2 shows that the Raman detection of RhB is strongly dependent on sputtering time, which means that there is a dependence on the density of distribution of silver clusters. Is this experimental observation coherent with the mechanism proposed? In other words, is polarizability affected by the nanoparticle size? This aspect is not clear, as nanoparticle size seems to provide a greater contribution to Raman enhancement.

Plasmonic optical field enhancement and hence SERS are indeed strongly controlled by nanoparticle size. However, the PIERS enhancement vs. SERS is robust for the same Ag nanoparticle size distributions. Using thermal dewetting, we can control the size distribution of Ag nanoparticles, which we show in SI Fig. S6, and we show spectra and enhancement factors of PIERS and SERS structures with different Ag sputtering times in Table S2. We see that the increase of PIERS signals relative to SERS is consistent across multiple samples with different size distributions.

5) Page 8: "The PIERS substrate showed a 580% enhancement of Raman signals than the equivalent SERS substrate without defective crystalline TiO2". How do you calculate it?

We calculated the PIERS enhancement in SI Section S1, and now expand further with a more robust estimation of peak areas instead of intensities as before, and obtain more accurate enhancement factors:

The Raman enhancement factor (EF) is calculated using Eqn (S1) below, via integrating the area of the peak beneath two different Raman bands (1648 cm^{-1} and 1358 cm^{-1}) to give I_{SERS} and I_{bulk}. The intensities are then scaled by the molar concentration of the RhB analyte in the case of the bulk powder v.s. the molecule of a certain concentration deposited on the SERS/PIERS substrates, which is a well-established method to estimate the EF[25, 26]. This EF is an underestimate as the likely concentration of the molecule is smaller than that shown as some molecules will not remain bound on the surface or within SERS hotspots.

$$\mathrm{EF} = \left(\frac{I_{SERS}/N_{SERS}}{I_{bulk}/N_{bulk}}\right) = \left(\frac{I_{SERS}/I_{bulk}}{C_{SERS}/C_{bulk}}\right) \quad \text{Eqn. (S1)}$$

Table S1: Calculation of the Enhancement Factor (EF)

Substrate	***Conc. RhB [M]***	***Peak area (cts.cm/mW.s)***		***EF***		
		1648 cm^{-1}	*1358 cm^{-1}*	*1648 cm^{-1}*	*1358 cm^{-1}*	*Average*
PIERS	10^{-5}	381548	492984	1.6×10^{6}	2.9×10^{6}	$2.3\times\mathbf{10^{6}}$
SERS	10^{-5}	73040	64171	3.1×10^{5}	3.7×10^{5}	$3.4\times\mathbf{10^{5}}$
Raman	2.75	64219	47272	1	1	1

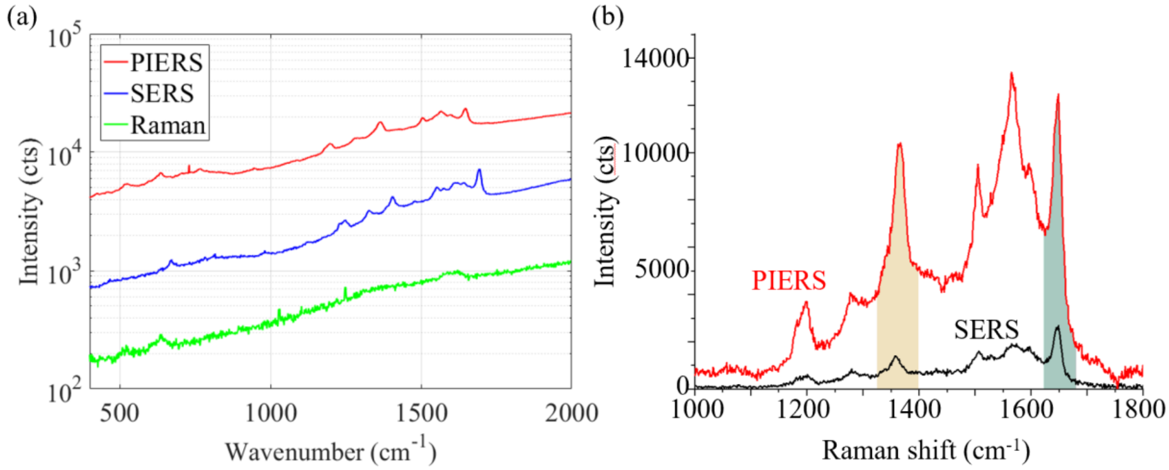

Figure R4 Raman spectra from powder, SERS, and PIERS substrates. (a) Non background corrected Raman spectra of RhB using PIERS, SERS, and no enhancement on aluminium foil (all at 10-5 M concentration of RhB) showing the increase in the fluorescence background. (b) Shaded integrated area taken for EF calculations centered at 1358 and 1648 cm^{-1}.

6) Introduction (page 2): ...”these techniques have not achieved comparable SERS enhancement or work as reproducibly as metal nanoparticle-based SERS”. This statement is questionable and somehow misleading. Literature of last ten years reports many examples showing that all-dielectric SERS active substrates are more reproducible and sensitive than plasmonic SERS (Chem. Rev 2016, J. Raman Spectrosc ; J. Raman Spectrosc.; Nat. Commun. 6, 7800 (2015)). Moreover, they are less invasive and allow for monitoring chemical reactions with minimal perturbation. (JACS 2013; Small 2014, Nature Commun. 2018)

We thank the reviewer for pointing out these articles – and have modified our introduction to cite several of these works. However, it is the case that the absolute field enhancements obtained by dielectric structures are lower as they do not support the plasmonic gap-modes required for single molecule fluctuation sensing[27] or single molecule strong coupling[28], and plasmonic nanogaps exceed most reported enhancement factors and are also highly reproducible[29]. But we do agree that more consideration needs to be given to dielectric media for abundance and low cost, which is of interest to the community, and we include a discussion in introduction, along with other low-cost plasmonic metals such as Mg and Al. We have corrected the introduction to highlight the reproducibility of dielectric structures, and the further possibilities achieved when combining them with noble metals:

“Many dielectrics can act as passive elements supporting plasmonic metal nanostructures to localize electromagnetic fields via a micro-lensing effect[5], and providing an inert shell that assists with in-situ SERS measurements (e.g. SHINERS[6] or SPARKs[7]). Semiconductors may also act as active SERS substrates via charge transfer to the target analyte molecule[8, 9] and form reusable SERS substrates

through photocatalytic degradation of adsorbed molecules[10] upon exposure to ultraviolet light and are abundant/low cost. These techniques have not achieved single-molecule SERS enhancements generally (EF = 10^2-10^6 [11]) or work as reproducibly as metal nanoparticle-based SERS[12]. Dielectric SERS[9] remains useful due to its abundance and lower cost, complementing abundant metals such as Mg[13] and Al[14], and its ability to interrogate chemical reactions[15]."

Editor comments:

1) Title: In both the main manuscript file and the Supporting Information, set the title in title case, with the first letter of each principal word capitalized.

2) Abstract: Shorten the abstract to 150 words or fewer.

3) Headers: Remove the section heading(s) throughout the body of the manuscript (you can leave Methods and Abstract headings).

4) TOC Graphic: Please resize the TOC graphic per journal guidelines (2 in x 2 in) and move to the correct position (on the same page as the abstract).

*5) References: In both the main file and the supporting information, fix the style of all references to use JPCL formatting (check all references carefully). ***JPC Letters reference formatting requires that journal references should contain: () around numbers, author names, article title (titles entirely in title case or entirely in lower case), abbreviated journal title (italicized), year (bolded), volume (italicized), and pages (first-last). Book references should contain author names, book title (in the same pattern), publisher, city, and year. Websites must include date of access.*

6) Supporting Information: Please number SI pages in the following format: "S1, S2…"

We thank the editor for pointing these out and have implemented all changes.

References

1. Ben-Jaber, S.; Peveler, W. J.; Quesada-Cabrera, R.; Cortés, E.; Sotelo-Vazquez, C.; Abdul-Karim, N.; Maier, S. A.; Parkin, I. P., Photo-induced enhanced Raman spectroscopy for universal ultra-trace detection of explosives, pollutants and biomolecules. *Nature Communications* **2016,** *7*, 12189.
2. Faried, M.; Suga, K.; Okamoto, Y.; Shameli, K.; Miyake, M.; Umakoshi, H., Membrane surface-enhanced Raman spectroscopy for cholesterol-modified lipid systems: effect of gold nanoparticle size. *ACS Omega* **2019,** *4* (9), 13687-13695.
3. Moskovits, M., Surface-enhanced spectroscopy. *Reviews of Modern Physics* **1985,** *57* (3), 783.
4. Busson, B.; Farhat, M.; Nini Teunda, P.-J.; Roy, S.; Jarisz, T.; Hore, D. K., All-experimental analysis of doubly resonant sum-frequency generation spectra: Application to aggregated rhodamine films. *The Journal of Chemical Physics* **2021,** *154* (22), 224704.
5. Turley, H. K.; Hu, Z.; Jensen, L.; Camden, J. P., Surface-enhanced resonance hyper-Raman scattering elucidates the molecular orientation of rhodamine 6G on silver colloids. *The Journal of Physical Chemistry Letters* **2017,** *8* (8), 1819-1823.
6. Bioquest, A., AAT Bioquest's Interactive Spectrum Viewer 2023.

7. Zheng, X.; Ren, F.; Zhang, S.; Zhang, X.; Wu, H.; Zhang, X.; Xing, Z.; Qin, W.; Liu, Y.; Jiang, C., A general method for large-scale fabrication of semiconducting oxides with high SERS sensitivity. *ACS Applied Materials & Interfaces* **2017,** *9* (16), 14534-14544.
8. Almohammed, S.; Zhang, F.; Rodriguez, B. J.; Rice, J. H., Photo-induced surface-enhanced Raman spectroscopy from a diphenylalanine peptide nanotube-metal nanoparticle template. *Scientific Reports* **2018,** *8* (1), 3880.
9. Feng, E.; Zheng, T.; He, X.; Chen, J.; Tian, Y., A novel ternary heterostructure with dramatic SERS activity for evaluation of PD-L1 expression at the single-cell level. *Science Advances* **2018,** *4* (11), eaau3494.
10. Yang, L.; Peng, Y.; Yang, Y.; Liu, J.; Huang, H.; Yu, B.; Zhao, J.; Lu, Y.; Huang, Z.; Li, Z., A Novel Ultra-Sensitive Semiconductor SERS Substrate Boosted by the Coupled Resonance Effect. *Advanced Science* **2019**, 1900310.
11. Wang, Y.; Liu, J.; Ozaki, Y.; Xu, Z.; Zhao, B., Effect of TiO2 on Altering Direction of Interfacial Charge Transfer in a TiO2-Ag-MPY-FePc System by SERS. *Angewandte Chemie* **2019**.
12. Hou, X.; Luo, X.; Fan, X.; Peng, Z.; Qiu, T., Plasmon-coupled charge transfer in WO 3− x semiconductor nanoarrays: toward highly uniform silver-comparable SERS platforms. *Physical Chemistry Chemical Physics* **2019,** *21* (5), 2611-2618.
13. Yang, L.; Sang, Q.; Du, J.; Yang, M.; Li, X.; Shen, Y.; Han, X.; Jiang, X.; Zhao, B., A Ag synchronously deposited and doped TiO 2 hybrid as an ultrasensitive SERS substrate: a multifunctional platform for SERS detection and photocatalytic degradation. *Physical Chemistry Chemical Physics* **2018,** *20* (22), 15149-15157.
14. Cong, S.; Yuan, Y.; Chen, Z.; Hou, J.; Yang, M.; Su, Y.; Zhang, Y.; Li, L.; Li, Q.; Geng, F., Noble metal-comparable SERS enhancement from semiconducting metal oxides by making oxygen vacancies. *Nature Communications* **2015,** *6*, 7800.
15. Li, M.; Fan, X.; Gao, Y.; Qiu, T., W18O49/monolayer MoS2 Heterojunction-Enhanced Raman Scattering. *The Journal of Physical Chemistry Letters* **2019**.
16. Muntwiler, M.; Lindstrom, C.; Zhu, X.-Y., Delocalized electron resonance at the alkanethiolate self-assembled monolayer⁄ Au (1 1 1) interface. *The Journal of Chemical Physics* **2006,** *124* (8), 081104.
17. Sotthewes, K.; Heimbuch, R.; Zandvliet, H. J., Manipulating transport through a single-molecule junction. *The Journal of Chemical Physics* **2013,** *139* (21), 214709.
18. Boerigter, C.; Aslam, U.; Linic, S., Mechanism of charge transfer from plasmonic nanostructures to chemically attached materials. *ACS Nano* **2016,** *10* (6), 6108-6115.
19. Kos, D.; Assumpcao, D. R.; Guo, C.; Baumberg, J. J., Quantum tunneling induced optical rectification and plasmon-enhanced photocurrent in nanocavity molecular junctions. *ACS Nano* **2021,** *15* (9), 14535-14543.
20. Glass, D.; Cortés, E.; Ben-Jaber, S.; Brick, T.; Peveler, W. J.; Blackman, C. S.; Howle, C. R.; Quesada-Cabrera, R.; Parkin, I. P.; Maier, S. A., Dynamics of Photo-Induced Surface Oxygen Vacancies in Metal-Oxide Semiconductors Studied Under Ambient Conditions. *Advanced Science* **2019,** *6* (22), 1901841.
21. Dong, J.; Ullal, R.; Han, J.; Wei, S.; Ouyang, X.; Dong, J.; Gao, W., Partially crystallized TiO 2 for microwave absorption. *Journal of Materials Chemistry A* **2015,** *3* (10), 5285-5288.
22. Luttrell, T.; Halpegamage, S.; Tao, J.; Kramer, A.; Sutter, E.; Batzill, M., Why is anatase a better photocatalyst than rutile?-Model studies on epitaxial TiO2 films. *Scientific Reports* **2014,** *4* (1), 4043.
23. Dong, J.; Han, J.; Liu, Y.; Nakajima, A.; Matsushita, S.; Wei, S.; Gao, W., Defective black TiO2 synthesized via anodization for visible-light photocatalysis. *ACS Applied Materials & Interfaces* **2014,** *6* (3), 1385-1388.
24. Jiang, X.; Zhang, Y.; Jiang, J.; Rong, Y.; Wang, Y.; Wu, Y.; Pan, C., Characterization of oxygen vacancy associates within hydrogenated TiO2: a positron annihilation study. *The Journal of Physical Chemistry C* **2012,** *116* (42), 22619-22624.

25. Le Ru, E.; Blackie, E.; Meyer, M.; Etchegoin, P. G., Surface enhanced Raman scattering enhancement factors: a comprehensive study. *The Journal of Physical Chemistry C* **2007,** *111* (37), 13794-13803.
26. Le Ru, E.; Etchegoin, P., *Principles of Surface-Enhanced Raman Spectroscopy: and related plasmonic effects*. Elsevier: 2008.
27. Kneipp, J.; Kneipp, H.; Kneipp, K., SERS—a single-molecule and nanoscale tool for bioanalytics. *Chemical Society Reviews* **2008,** *37* (5), 1052-1060.
28. Chikkaraddy, R.; De Nijs, B.; Benz, F.; Barrow, S. J.; Scherman, O. A.; Rosta, E.; Demetriadou, A.; Fox, P.; Hess, O.; Baumberg, J. J., Single-molecule strong coupling at room temperature in plasmonic nanocavities. *Nature* **2016,** *535* (7610), 127-130.
29. Grys, D. B.; Chikkaraddy, R.; Kamp, M.; Scherman, O. A.; Baumberg, J. J.; de Nijs, B., Eliminating irreproducibility in SERS substrates. *Journal of Raman Spectroscopy* **2021,** *52* (2), 412-419.

jz-2023-00478r.R2

Name: Peer Review Information for "Understanding the Chemical Mechanism Behind Photo-Induced Enhanced Raman Spectroscopy"

Second Round of Reviewer Comments

Reviewer: 1

Comments to the Author

I am satisfied with the authors reply and modifications to the manuscript.

A careful checking of the manuscript for typo's and grammatical errors is still recommended as I found numerous problems like this upon my rereading.

Author's Response to Peer Review Comments:

Response to reviewers

We thank the editor and reviewers for the chance to address comments again in this round of review, and believe our final minor revisions regarding the manuscripts presentation addresses all concerns. We thank the first reviewer for highlighting that the scholarly presentation, the urgency, the significance, and general interest regarding this paper's conclusions is high.

Reviewer #1:

"Recommendation: This paper is publishable subject to minor revisions noted. Further review is not needed."

We thank the reviewer for their efforts in improving this manuscript's presentation.

"Comments: I am satisfied with the authors reply and modifications to the manuscript. A careful checking of the manuscript for typos and grammatical errors is still recommended as I found numerous problems like this upon my rereading."

We have gone through the manuscript and corrected any final typos and grammatical errors.

Editor comments:

1) Highlighting: Please remove the colored text or highlights showing the changes made to the manuscript and Supporting Information files. Please upload "clean" copies for publication. You may upload annotated files separately as "Supporting Information for Review Only" files.
2) Headers: Remove the section heading(s) throughout the body of the manuscript (you can leave Methods and Abstract headings).

We thank the editor for pointing these out and have implemented all changes.
